# Steric hindrance, ligand ejection and associated photocytotoxic properties of ruthenium(II) polypyridyl complexes

**DOI:** 10.1007/s00775-023-01998-z

**Published:** 2023-04-15

**Authors:** Piedad Herrera-Ramírez, Sarah Alina Berger, Dana Josa, David Aguilà, Ana B. Caballero, Pere Fontova, Vanessa Soto-Cerrato, Manuel Martínez, Patrick Gamez

**Affiliations:** 1 Departament de Química Inorgànica i Orgànica, Facultat de Química, Secció de Química Inorgànica, Universitat de Barcelona, Martí i Franquès, 1-11, 08028 Barcelona, Spain; 2 Institute of Nanoscience and Nanotechnology (IN2UB), Universitat de Barcelona, Barcelona, Spain; 3 Department of Pathology and Experimental Therapeutics, Faculty of Medicine and Health Sciences, Universitat de Barcelona, Campus Bellvitge, Feixa Llarga s/n, 08907 L’Hospitalet de Llobregat, Barcelona Spain; 4 Department of Chemistry, Universidad de Burgos, 09001 Burgos, Spain; 5 Oncobell Program, Institut d’Investigació Biomèdica de Bellvitge (IDIBELL), L’Hospitalet de Llobregat, Barcelona, Spain; 6 Catalan Institution for Research and Advanced Studies (ICREA), Passeig Lluís Companys 23, 08010 Barcelona, Spain

**Keywords:** Ruthenium(II), Photochemistry, Ligand photorelease, Photoactivated chemotherapy, Light activation, Photoreaction

## Abstract

Two ruthenium(II) polypyridyl complexes were prepared with the {Ru(phen)_2_}^2+^ moiety and a third sterically non-hindering bidentate ligand, namely 2,2′-dipyridylamine (dpa) and *N*-benzyl-2,2′-dipyridylamine (Bndpa). Hence, complexes [Ru(phen)_2_(dpa)](PF_6_)_2_ (**1**) and [Ru(phen)_2_(Bndpa)](PF_6_)_2_ (**2**) were characterized and their photochemical behaviour in solution (acetonitrile and water) was subsequently investigated. Compounds **1** and **2**, which do not exhibit notably distorted octahedral coordination environments, contrarily to the homoleptic “parent” compound [Ru(phen)_3_](PF_6_)_2_, experience two-step photoejection of the dpa and Bndpa ligand upon irradiation (1050–430 nm) for several hours. DNA-binding studies revealed that compounds **1** and **2** affect the biomolecule differently upon irradiation; while **2** solely modifies its electrophoretic mobility, complex **1** is also capable of cleaving it. In vitro cytotoxicity studies with two cancer-cell lines, namely A549 (lung adenocarcinoma) and A375 (melanoma), showed that both **1** and **2** are not toxic in the dark, while only **1** is significantly cytotoxic if irradiated, **2** remaining non-toxic under these conditions.

**Graphical abstract:**

Light irradiation of the complex cation [Ru(phen)_2_(dpa)]^2+^ leads to the generation of transient Ru species that is present in the solution medium for several hours, and that is significantly cytotoxic, ultimately producing non-toxic free dpa and [Ru(phen)(OH_2_)_2_]^2+^.

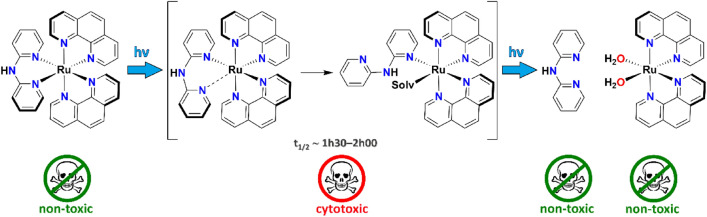

**Supplementary Information:**

The online version contains supplementary material available at 10.1007/s00775-023-01998-z.

## Introduction

The impact of cancer on society is major as it currently represents the second leading cause of death worldwide [1]. Chemotherapy is one of the most common types of cancer treatment [2]. Nevertheless, it often suffers from some severe and unpleasant side effects [3], as observed with the well-known chemotherapeutic drug cisplatin [4], whose efficacy can even be reduced leading to platinum-resistant tumours [5]. The development of more selective and efficient anticancer agents to overcome adverse effects and drug resistance is therefore a topical field of research [6, 7]. Various approaches may be applied to improve the selectivity and efficiency of chemical drugs, such as the use of targeting groups [8], nanoparticles [9], stimuli-responsive drug-delivery systems [10] or stimuli-activatable prodrugs (*e.g.*, activation through pH, light, sound, redox environment, etc.) [11–14]. Due to the possibility to switch them on in a controllable manner, photoactivatable compounds are currently experiencing a great deal of attention from the scientific community, mostly for antibacterial [15], dermatologic [16] or anticancer [11] applications. The increasing interest in the possible use of photoresponsive, metal-based anticancer drugs stems from the highly promising properties of TLD-1433, a polypyridyl Ru(II) complex reported by McFarland, Lilge and co-workers [1], which has entered Phase II clinical trials for the treatment of non-muscle invasive bladder cancer (NMIBC) [2]. Hence, numerous transition-metal-containing photosensitizers have been described in the literature for potential utilization in photodynamic therapy (PDT) and photoactivated chemotherapy (PACT) [17–19].

PDT requires the simultaneous presence of three fundamental components, namely a photosensitizer (PS), molecular oxygen and light [20]. Upon irradiation, the PS is promoted to an excited singlet state (PS_Es_), which can convert to a long-lived triplet excited state (PS_Et_) via intersystem crossing (ISC). Two types of quenching reactions can then occur: (i) *type I reactions* where PS_Et_ reacts with biomolecules through hydrogen (electron) transfer, generating radicals that subsequently react with molecular oxygen to produce reactive oxygen species (ROS), such as HO^·^, H_2_O_2_ and O_2_^·−^; (ii) *type II reactions* where PS_Et_ directly reacts with ground state triplet molecular oxygen (^3^O_2_), generating singlet oxygen (^1^O_2_) via energy transfer [21]. The ROS generated in situ are extremely harmful and will induce severe cellular damages, resulting in necrosis, apoptosis, autophagy and/or immunogenic cell death [22, 23]. *Type II reactions* are generally considered as the main photosensitization mechanism in PDT [24].

In PACT, cytotoxic species are generated in a controlled manner through light irradiation of inactive metal complexes [17]. Four types of PACT can be defined, based on their respective mechanism of action: (i) *Photoinduced electron transfer* from ligand to metal may give rise to the release of the (active) ligand and the generation of toxic reduced metal species [25] the alternative metal oxidation may also occur producing highly oxidizing species altering biomolecules like DNA [26]; (ii) *Photosubstitution* for which light irradiation produces an excited metal-to-ligand charge-transfer triplet state that rapidly interconverts into a highly dissociative excited metal-centred triplet state, giving rise to ligand release and the generation of very reactive species [27]; (iii) *Bioactive ligand release*, *e.g.* NO or CO, by metal complexes upon light irradiation [28]; (iv) *Ligand photocleavage* including the release (photocleavage) or formation (photoswitch) of active species [29, 30].

Polypyridyl ruthenium(II) compounds represent a highly promising family of photoactive complexes; they are stable under both dark and light conditions, exhibit strong light absorption and efficiently mediate the generation of singlet dioxygen [1, 31]. For instance, the ruthenium(II) complex TLD-1433 has a remarkably high quantum yield of ^1^O_2_ production and is therefore highly photocytotoxic toward cancer cells [32, 33].

Most of the studied ruthenium-based PACT compounds originate from [Ru(bpy)_3_]^2+^ (bpy = 2,2′-bipyridine) where one of the bpy ligands is substituted by a *sterically hindered* heteroaromatic *N*,*N*-donor ligand (**L**), such as 6,6′-dimethyl-2,2′-bipyridine (dmbpy) [34, 35]. It is believed that the cytotoxic activity observed arises from the formation of the complex [Ru(bpy)_2_(OH_2_)_2_]^2+^ upon photorelease of dmbpy [35]. [Ru(bpy)_2_(OH_2_)_2_]^2+^ has also been proposed as the active species for other [Ru(bpy)_2_**L**]^2+^-type complexes [36, 37]. However, some other studies suggested that [Ru(bpy)_2_(OH_2_)_2_]^2+^ is not toxic [38, 39]; for example, it has been shown that the photoreleased dmbpy ligand is already 25 times more cytotoxic than the original [Ru(bpy)_2_(dmbpy)]^2+^ complex against A549 cells (human lung adenocarcinoma) [27]. Similarly, [Ru(phen)_2_(OH_2_)_2_]^2+^ (phen = 1,10-phenanthroline) has been described as photogenerated cytotoxic species for [Ru(phen)_2_**L**]^2+^-type complexes [27, 40, 41].

In the present study, two heteroleptic polypyridyl ruthenium(II) complexes of formula [Ru(phen)_2_(**L**)]X_2_ have been prepared (L = dpa, Bndpa; Fig. S1) with the objective to investigate its photochemical behaviour, interaction with DNA and in vitro cytotoxicity. Hence, [Ru(phen)_2_(dpa)](PF_6_)_2_ (**1**) was typically obtained by reaction of the well-known complex precursor [Ru(phen)_2_Cl_2_] with the flexible ligand 2,2′-dipyridylamine (dpa). Furthermore, a slightly modified complex, namely [Ru(phen)_2_(Bndpa)](PF_6_)_2_ (**2**) (where Bndpa stands for *N*-benzyl-2,2′-dipyridylamine), was synthesized to assess the potential effect of the benzylation of the ligand on the photochemical and in vitro biological properties.

## Experimental

### General considerations

All reactions were carried out under an inert atmosphere of dinitrogen using standard Schlenk techniques. Nuclear magnetic resonance (NMR) spectra were recorded in CD_3_CN at room temperature on a Varian Mercury 400 spectrometer (^1^H NMR) or Bruker Avance III 400 spectrometer (^13^C{^1^H} NMR and HSQC). The chemical shifts (*δ*) are reported in parts per million (ppm) and are referenced to the nondeuterated solvent residual peak (CH_3_CN: 1.94 ppm for ^1^H spectra, 1.3 and 118.3 ppm for ^13^C spectra). All NMR data were analyzed using MestRe Nova Version 14.2.1 [42]. Mass spectrometry analyses (MS) were carried out with a Waters ZQ 2000 spectrometer using electrospray ionization. UV/Vis spectra were recorded using a Varian Cary 100 scan spectrometer with a 1 cm path length quartz cuvette. Fluorescence measurements were performed on a HORIBA Jobin–Yvon iHR320 fluorometer with a 1 cm path-length quartz cuvette. Circular dichroism (CD) spectra were recorded with a JASCO J-815 CD spectropolarimeter using a 5 mm path-length quartz cuvette. X-ray structural determinations were performed on a Bruker APEX II QUAZAR diffractometer equipped with a microfocus multilayer monochromator with Mo Kα radiation (*λ* = 0.71073 Å). Data reduction and absorption corrections were performed using SAINT and SADABS, respectively [43]. The structure was solved using SHELXT [44] and refined with full-matrix least-squares on *F*^2^ by using SHELXL-2014 [45]. Photoactivation was achieved using an ASAHI spectra Max-303 300 W Xenon lamp (output wavelength: 250–1050 nm) with a cut-off filter for wavelength below 430 nm. All plots were created using RStudio Version 1.4.1106 [46].

### Materials

DMSO, acetonitrile, sodium cacodylate, sodium azide, NH_4_PF_6_ and NaCl were purchased from Fisher Scientific. Dichloromethane was purchased from Honeywell. Methanol, *N*,*N*-dimethylformamide, ethanol, 2,2′-dipyridylamine (dpa), calf thymus DNA, Hoechst 33258 and 3-(4,5-dimethylthiazol-2-yl)-2,5-diphenyltetrazolium bromide (MTT) were purchased from Sigma-Aldrich. Diethylether, benzyl bromide and 1,10-phenanthroline were purchased from ACROS Organics. RuCl_3_·3H_2_O was purchased from Pressure Chemical. KOH and LiCl were obtained from Panreac and hydroquinone (viz. benzene-1,4-diol) from Riedel-de Haën. pBR322 plasmid was purchased from ThermoFisher Scientific. SYBR safe and 10 × TBE buffer were purchased from Invitrogen. Agarose was obtained from Ecogen. All solvents and reagents were used without further purification.

### Synthetic procedures

*N*-Benzyldi(2-pyridyl)amine (Bndpa). Bndpa was synthesized as reported in the literature [47]. 0.53 g (9.45 mmol) of KOH and 0.4 g (2.34 mmol) of 2,2′-dipyridylamine (dpa) were dissolved in 4 mL of DMSO in a two-necked round-bottomed flask of 25 mL, under an inert atmosphere of dinitrogen. The resulting reaction mixture was stirred for 40 min at room temperature. 0.278 mL (2.34 mmol; 0.4 g) of benzyl bromide was subsequently added and the reaction mixture was stirred for 24 h at room temperature. 5 mL of deionized water were then added, and the solution was kept in the fridge overnight. The yellow–brown solid obtained was isolated by filtration and washed three times (3 × 10 mL) with deionized water. The crude compound was purified by column chromatography on silica gel using dichloromethane:methanol 9:1 as the eluent. Pure Bndpa was obtained as a white solid with a yield of 58% (0.36 g; 1.36 mmol). C_17_H_15_N_3_, M_w_ = 261.33 g mol^−1^. ^1^H NMR (400 MHz, CD_3_CN): *δ* 8.25 (ddq, *J* = 7.1, 4.9, 0.8 Hz, 2H), 7.58 (dddd, *J* = 9.1, 7.2, 2.0, 0.7 Hz, 2H), 7.33 (dtd, *J* = 6.9, 1.5, 0.7 Hz, 2H), 7.30—7.13 (m, 5H), 6.90 (ddt, *J* = 7.3, 4.9, 0.8 Hz, 2H), 5.45 (s, 2H) ppm (Fig. S2). LRMS (ES +) m/z [M + H]^+^ calcd. for C_17_H_16_N_3_, 262.34; found, 262.16 (Fig. S3).*cis*-Dichlorobis(1,10-phenanthroline)ruthenium(II) (**[Ru(phen)**_**2**_**Cl**_**2**_**]**). This precursor compound was prepared following a synthetic procedure described in the literature for a related ruthenium(II) compound [48]. 0.5 g (2.4 mmol) of anhydrous RuCl_3_ (RuCl_3_·3H_2_O dried in an oven at 80 °C for several days) were suspended in 12 mL of DMF under an atmosphere of N_2_. 0.53 g (4.8 mmol, 2 eq.) of hydroquinone and 0.53 g (12 mmol, 5 eq.) of LiCl were subsequently added. The mixture was stirred for 15 min before the addition of 0.86 g (4.8 mmol, 2 eq.) of 1,10-phenanthroline (phen). The resulting reaction mixture was refluxed for 1 h and poured into 250 mL of distilled water. The black precipitate formed was isolated by filtration. It was dissolved in hot DMF and re-precipitated in 250 mL of distilled water. This step was performed twice, and the final precipitate was washed with EtOH and Et2O. **[Ru(phen)**_**2**_**Cl**_**2**_**]** was obtained with a yield of 60% (0.76 g; 1.44 mmol). It was stored at room temperature, protected from light. C_24_H_16_Cl_2_N_4_Ru, M_w_ = 532.39 g mol^−1^. ^1^H NMR (400 MHz, CD_3_CN): *δ* 10.28 (d, *J* = 4.0 Hz, 2H), 8.72 (d, *J* = 4.0 Hz, 2H), 8.30 (d, *J* = 4.0 Hz, 2H), 8.25–8.21 (m, 4H), 8.15 (d, *J* = 4.0 Hz, 2H), 7.76 (d, *J* = 4.0 Hz, 2H), 7.34 (dd, *J* = 4.0 Hz, *J* = 4.0 Hz, 2H) ppm (Fig. S4). LRMS (ES +) m/z [M]^+^ calcd. for C_24_H_16_Cl_2_N_4_Ru, 531.98; found, 531.97; m/z [M-Cl]^+^ calcd. for C_24_H_16_ClN_4_Ru, 497.01; found, 496.90; m/z [M + EtOH]^+^ calcd. for C_26_H_22_Cl_2_N_4_ORu, 578.02; found, 577.99 (Fig. S5).

Bis(1,10-phenanthroline)-(2,2′-dipyridylamine)ruthenium(II) bis(hexafluorophosphate) (**1**)*.* 0.20 g (0.38 mmol) of **[Ru(phen)**_**2**_**Cl**_**2**_**]** was dissolved in 15 mL of ethanol in a two-necked round-bottomed flask of 50 mL under an inert atmosphere of N_2_. 0.065 g (0.38 mmol, 1 eq.) of ligand 2,2´-dipyridylamine (dpa) was added and the reaction mixture was refluxed for 24 h. The remaining black precipitate of **[Ru(phen)**_**2**_**Cl**_**2**_**]** was removed by filtration and 10 drops of a saturated aqueous solution of NH_4_PF_6_ were added to the filtrate under stirring. The resulting red precipitate was isolated by filtration and washed with EtOH (2 × 10 mL) and Et_2_O (2 × 10 mL). The crude was purified by column chromatography on silica gel using as an eluent a 9:1 mixture of acetonitrile and aqueous KNO_3_ (0.1 M). The fraction corresponding to complex **1** was evaporated and the solid obtained was redissolved in acetonitrile; the white solid corresponding to KNO_3_ was filtered off, washed with diethyl ether and the solvent was removed under reduced pressure, giving pure **1** as a red solid with a yield of 43% (0.076 g, 0.12 mmol). Compound **1** was stored at room temperature, protected from light. The TLC of pure **1** (eluent: CH_3_CN 9/0.1 M KNO_3_ 1) is shown in Fig. S6. Single crystals of **1**, suitable for X-ray diffraction analysis, were obtained by slow diffusion of diethyl ether into a saturated solution of the ruthenium complex in acetonitrile; an X-ray structure of **1** was reported in 2008 [49]. C_34_H_25_F_12_N_7_P_2_Ru, M_w_ = 922.62 g mol^−1^. ^1^H NMR (400 MHz, CD_3_CN): *δ* 10.65 (1H, NH), 9.04 (dd, *J* = 8, 0.4 Hz, 2H), 8.76 (dd, *J* = 8, 0.4 Hz, 2H), 8.44 (dd, *J* = 9, 0.4 Hz, 2H), 8.29 (d, *J* = 8 Hz, 2H), 8.18 (d, *J* = 8 Hz, 2H), 8.02 (dd, *J* = 8, 4 Hz, 2H), 7.68 (dd, 8, 0.4 Hz, 2H), 7.66–7.63 (m, 4H), 7.42 (dd, *J* = 9, 8 Hz, 2H), 7.14 (d, *J* = 8 Hz, 2H), 6.55 (dd, *J* = 8, 8 Hz, 2H) ppm (Fig. S7). ^13^C NMR (101 MHz, CD_3_CN): *δ* 154.3, 153.9, 152.8, 150.2, 148.3, 147.9, 138.4, 136.9, 136.2, 131.2, 131.0, 128.2, 127.9, 125.7, 118.8, 114.9 ppm (Fig. S8). The NMR peaks were unambiguously assigned by using the ^1^H-^13^C HSQC NMR spectrum of **1** (Fig. S9). LRMS (ES +) m/z [M]^2+^ calcd. for C_34_H_25_N_7_Ru, 316.35; found, 316.47; m/z [M]^+^ calcd. for C_34_H_25_N_7_Ru, 632.69; found, 632.26 (Fig. S10). Anal. Calcd for C_38_H_35_F_12_N_7_OP_2_Ru [**1** + Et_2_O]: C, 45.79%; H, 3.54%; N, 9.84. Found: C, 46.05%; H, 3.38%; N, 10.45%. ESI-TOF (+) m/z [Ru(phen)_2_(dpa)]^2+^, 316.5781; found: 316.5606; {[Ru(phen)_2_(dpa)]—H}^+^, 632.1106; found: 632.1138.

Bis(1,10-phenanthroline)-(N-benzyldi(2-pyridyl)amine)ruthenium(II) bis(hexafluorophosphate) (**2**)*.* 0.2 g (0.38 mmol) of **[Ru(phen)**_**2**_**Cl**_**2**_**]** was dissolved in 20 mL of ethanol in a two-necked bottomed flask of 50 mL under an inert atmosphere of N_2_. 0.1 g (0.38 mmol, 1 eq.) of ligand Bndpa was added and the reaction mixture was refluxed for 48 h. The black precipitate was eliminated by filtration and 15 drops of a saturated aqueous solution of NH_4_PF_6_ were added to the filtrate under stirring. The resulting orange precipitate was isolated by filtration and washed with EtOH (2 × 10 mL) and Et_2_O (2 × 10 mL). [Ru(phen)_2_(Bndpa)](PF_6_)_2_ (**2**) was obtained as an orange solid with a yield of 26% (0.1 g, 0.10 mmol). Compound **2** was stored at room temperature, protected from light. Single crystals of **2**, suitable for X-ray diffraction analysis, were obtained by slow diffusion of diethyl ether into a saturated solution of the ruthenium complex in acetonitrile. C_41_H_31_F_12_N_7_P_2_Ru, M_w_ = 1012.75 g mol^−1^. ^1^H NMR (400 MHz, CD_3_CN): *δ* 8.92 (dd, *J* = 5.3, 1.3 Hz, 2H), 8.79 (dd, *J* = 8.2, 1.3 Hz, 2H), 8.47 (dd, *J* = 8.3, 1.3 Hz, 2H), 8.34 (d, *J* = 8.9 Hz, 2H), 8.22 (d, *J* = 8.9 Hz, 2H), 8.03 (dd, *J* = 8.2, 5.3 Hz, 2H), 7.74 (dd, *J* = 5.3, 1.3 Hz, 2H), 7.71–7.63 (m, 2H), 7.56–7.43 (m, 4H), 7.34–7.20 (m, 3H), 7.16 (tt, *J* = 6.8, 1.6 Hz, 2H), 6.85–6.68 (m, 4H), 5.52 (d, *J* = 17.3 Hz, 1H), 5.11 (dd, *J* = 17.3, 1.2 Hz, 1H) ppm (Fig. S11). ^13^C NMR (101 MHz, CD_3_CN): *δ* 158.7, 154.8, 153.7, 152.3, 149.3, 148.9, 139.8, 138.0, 137.2, 135.9, 132.3, 132.0, 129.6, 129.1, 129.0, 128.3, 127.4, 126.8, 126.6, 121.6, 117.9, 57.8 ppm (Fig. S12). The NMR peaks were unambiguously assigned by using the ^1^H-^13^C HSQC NMR spectrum of **2** (Fig. S13). LRMS (ES +) m/z [M]^2+^ calcd. for C_41_H_31_N_7_Ru, 361.41; found, 361.58; m/z [M-Bn]^2+^ calcd. for C_34_H_24_N_7_Ru, 316.08; found, 315.94; m/z [M-Bn]^+^ calcd. for C_34_H_24_N_7_Ru, 631.69; found, 632.23 (Fig. S14). Anal. Calcd for C_38_H_35_F_12_N_7_OP_2_Ru [**2** + 2 H_2_O]: C, 46.95%; H, 3.36%; N, 9.35. Found: C, 46.74%; H, 3.37%; N, 9.00%. ESI-TOF (+) m/z [Ru(phen)_2_(Bndpa)]^2+^, 361.5889; found: 361.5842. Bn stands for benzyl; Bndpa appears to be debenzylated upon ionization in the mass spectrometer and is therefore converted to dpa (see “Behaviour of 1 and 2 upon irradiation in solution” section).

### X-ray crystallography.

Data for compound** 2** were collected on a Bruker APEX II QUAZAR diffractometer equipped with a microfocus multilayer monochromator with MoKα radiation (λ = 0.71073 Å). Data reduction and absorption corrections were performed by using SAINT and SADABS, respectively [43]. The structures were solved using SHELXT [44] and refined with full-matrix least squares on *F*^2^ by using SHELXL-2014 [45]. All details can be found in CCDC **2218777** that contain the supplementary crystallographic data for this paper. These data can be obtained free of charge from The Cambridge Crystallographic Data Center via https://summary.ccdc.cam.ac.uk/structure-summary-form.

### Photochemistry

The behaviour of **1** and **2** upon irradiation with light in the 1050–430 nm wavelength range was studied using a xenon light source MAX*-*303 from Asahi Spectra (distance of the lamp: 10 cm; 6.5 mW cm^–2^). 50 µM solutions of the complexes in water and acetonitrile, unless stated. UV/Vis and mass spectra were recorded just before irradiation and after selected time intervals of irradiation. For the kinetic studies, UV/Vis spectra were recorded every at fixed intervals for several hours, using a 1 cm path-length quartz cuvette and an Agilent HP 8453A spectrometer equipped with a diode array detector. Irradiation (at 1050–430 nm) and measurement was conducted at different positions of a thermostated multicell transport system. The values of the rate constants and kinetic speciation profiles were performed by global analysis using the SPECFIT software [50]. All data were globally fitted to a one- and two-step process, and the best fit for the reaction investigated was hence determined. Mass spectra were recorded with an Agilent Technologies G1969A ESI-TOF spectrometer (using different ionization voltages) at multiple time points throughout the irradiation study in water to potentially identify reaction intermediates. NMR spectra were also recorded during the irradiation in acetonitrile on a Bruker Avance III 400 spectrometer.

### Lipophilicity

The lipophilicity of **1** and** 2** was quantified by calculating the partition coefficients in an octan-1-ol/water system using the "shake-flask" method. The complexes were suspended in milliQ water saturated with octan-1-ol. After sonicating them for 1 h at 298 K, the suspensions were shaken for 24 h using an orbital-shaker at a rate of 120 r.p.m. The samples were subsequently filtered with a 0.2 μm Puradisc FP 30 mm Cellulose Acetate Syringe Filter (Whatman). Some aliquots (of 4 mL) of the filtrates (fs samples) were reserved (for UV–Vis measurements). Other aliquots of 5 mL were poured onto 5 mL of octan-1-ol saturated with milliQ water. The resulting mixtures were shaken for 24 h at 298 K. The samples were then centrifuged, and the organic phases were isolated (cs samples). UV–Vis spectra were recorded for both the fs and cs samples (see Figs. S15 and S16). The observed differences between the MLCT absorptions of the two types of samples, namely A_fs_ and A_cs_, were used to calculate the log *P*_o/w_ values applying Eq. (3) (see main text). The data obtained after measurements in triplicate are listed in Table S1.

### DNA interaction studies

#### Gel electrophoresis

Electrophoretic experiments with pBR322 plasmid DNA were performed in cacodylate buffer (10 mM sodium cacodylate and 50 mM NaCl in milliQ water). Stock solutions of the complexes in DMSO at concentrations at least 200 times higher than that used in the gel electrophoresis experiments were prepared and diluted in cacodylate buffer. pBR322 was diluted in cacodylate buffer to obtain a DNA concentration of 15 µM (in base) and incubated with distinct concentrations of complexes, from 1.2 to 50 µM. Four different sample preparations (symbolized as P1–P4) were used, namely (1) dark control: the complex and DNA were incubated for 30 min at 37 °C and kept in the dark at room temperature (P1); (2) preirradiated sample: the complex solution was irradiated for 30 min with 1050–430 nm light (Max-303; Asahi Spectra) before the addition of DNA, followed by incubation at 37 °C for 30 min (P2); (3) irradiated sample: a solution of the complex and DNA was incubated for 30 min at 37 °C and subsequently irradiated for 30 min with 1050–430 nm light (P3); preirradiated/irradiated sample: the solution of the complex was irradiated for 30 min with 1050–430 nm light and DNA was then added. The resulting mixture was incubated for 30 min at 37 °C and subsequently irradiated with 1050–430 nm light for 30 min (P4). The P1–P4 procedure was also applied to carry out experiments with different preirradiation, irradiation and incubation times. Furthermore, the potential effect of radical quenchers was investigated with samples containing 10% DMSO or 5 mM NaN_3_. All samples were mixed with loading buffer (viz*.* xylene cyanol 0.25% aqueous solution containing 30% glycerol) and loaded onto 1% agarose gels (1% in TBE buffer). The gels were run for 1 h at 100 V using a Bio-Rad horizontal tank connected to a Consort EV231 variable potential power supply, and the DNA was stained with SYBR™ Safe overnight. All gels were imaged with a BioRad GelDoc EZ Imager.

#### UV/Vis binding studies

The interaction binding of **1** and **2** was investigated by UV/Vis spectroscopy, where absorption changes may give information about the DNA binding mode(s). Furthermore, the affinity of the complexes for the biomolecule can be evaluated applying Eq. (1) [51]:

1 $$\frac{[DNA]}{{\varepsilon }_{a}- {\varepsilon }_{f}}=\frac{[DNA]}{{\varepsilon }_{b}- {\varepsilon }_{f}}+ \frac{1}{{K}_{b}} \times \frac{1}{{\varepsilon }_{b}- {\varepsilon }_{f}}$$

[DNA] = concentration of DNA in base, *ε*_a_ = apparent extinction coefficient obtained from A_obs_/[complex], *ε*_f_ = extinction coefficient of the DNA-free complex solution, *ε*_b_ = extinction coefficient of the DNA-bound complex solution, *K*_b_ = intrinsic binding constant.

Calf-thymus DNA (ct-DNA) was dissolved in cacodylate buffer, and the solution was stirred overnight at room temperature. 25 µM solutions of complex were incubated for 1 h at 37 °C with increasing concentrations of ct-DNA, namely from 0.2 to 12 µM (in base). The absorption changes were measured by UV/Vis spectroscopy for the dark and preirradiated conditions. Dark conditions: the complex solution was kept in the dark before and after incubation with DNA for 1 h at 37 °C; preirradiated conditions: the complex solution was irradiated for 1 h with 1050–430 nm light before the addition of DNA and subsequent incubation for 1 h at 37 °C.

#### Displacement of Hoechst 33258

*Hoechst 33258 is a* minor groove DNA binder that fluoresces upon interaction with the biomolecule. The fluorescence quenching resulting from its displacement can be followed by fluorescence spectroscopy, and the Stern–Volmer constant, *i.e. K*_SV_, can be determined applying Eq. (2) [52]:

2 $$\frac{{I}_{0}}{I}=1- {K}_{SV}[L]$$

*I*_0_ = fluorescence intensity of *Hoechst 33258* bound to DNA, *I* = fluorescence intensity upon the addition of each concentration of compound (*i.e.*, the quenching molecule *L*), *K*_SV_ = quenching constant.

A solution of 30 µM DNA (in base) and 2 µM *Hoechst 33258* was incubated for 30 min at 37 °C to allow the minor groove binding of the dye. Complex solutions were either kept in the dark or irradiated for 4 h with 1050–430 nm light prior to their addition to the DNA-*Hoechst 33258* solution. Increasing amounts of complex, using concentrations from 0 to 50 µM, were added and the resulting samples were incubated for 30 min at 37 °C. Fluorescence emission spectra of *Hoechst 33258 (λ*_exc_ = 350 nm; *λ*_em_ = 458 nm) were registered after each incubation period and smoothed using a Savitzky-Golay filter [53].

### Circular dichroism

The B form of DNA has a well-known CD spectrum characterized by a maximum at 275 nm and a minimum at 245 nm [54]. Characteristic deviation(s) of this spectrum can be observed upon distortion of the DNA through its interaction with a molecule [55].

A 50 µM (in base) solution of ct-DNA was incubated for 1 h at 37 °C with increasing concentrations of complex, namely from 5 to 50 µM. Complex solutions were either kept in the dark or irradiated for 4 h with 1050–430 nm light prior to their addition to DNA. The CD spectra were recorded with a JASCO J-815 CD spectropolarimeter using the following parameters: sensitivity: high; band width: 1 nm; data pitch: 0.5 nm; scanning speed: 500 nm/min; accumulations: 5. Baseline correction was performed on all spectra, which were also smoothed using a Savitzky-Golay filter [53]. Additional experiments were performed with a DNA-complex incubation time of 24 h.

### Cytotoxicity

#### Cell lines and culture conditions

The A549 (human lung adenocarcinoma) and A375 (human melanoma) cell lines were purchased from the American Type Culture Collection (ATCC, Manassas, VA, USA). The two cell lines were tested and verified by ATCC using short tandem repeat analysis. They were cultured between passage number 10–25 and were routinely tested for mycoplasma contamination detection. The cells were cultured in high glucose Dulbecco’s Modified Eagle’s Medium (DMEM) medium (Biological Industries, 01–055-1A) supplemented with 10% heat-inactivated fetal bovine serum (FBS; Gibco 1027–106), 2 mM L-glutamine, (Gibco 03–020-1B), 100 unit/mL penicillin and 100 μg/mL streptomycin (Gibco 03–031-1B). The cell lines were grown at 37 °C under a humid atmosphere containing 5% CO_2_.

#### Cell viability assays

Cell proliferation was evaluated by the MTT assay. The cells (1 × 10^4^ cells per mL) were plated in 96-well sterile plates and allowed to grow for 24 h. After attachment to the surface, the cells were subsequently incubated at 37 °C with decreasing concentrations of the complexes, from 100 to 0.8 µM; the complex solutions were freshly prepared from stock solutions in DMSO and diluted in the culture medium (the final concentration of DMSO was not exceeding 1%). Control cells were cultured as well in the same culture medium plus the carrier, viz. 1% DMSO. Compounds **1** and **2** were incubated with cells for 1 h at 37 °C to allow cellular uptake under both conditions. For the “pre-irradiation conditions”, the cells were then irradiated for 1 h at room temperature with a home-made lamp (Fig. S17) made of 96 white LEDs (RND components, ref.: RND 135-00220 purchased from DISTRELEC; spectral distribution, see Fig. S18), connected to an APELEX ST305 DC power supply; parameters: voltage = 288 V, current = 25 mA, luminous intensity = 8.5 cd per LED, color temperature = 5500 K, distance between the LEDS and the wells from the cell culture plate = 6.2 cm (Fig. S17). For the “dark conditions”, the cells were then incubated for 1 h at room temperature but keeping the cell culture plates protected from light. Following the incubation for 48 h, 10 μM MTT were added to each well, and the cells were further incubated for 2 h to allow the metabolization of MTT by living cells, converting it into its insoluble formazan form. The medium was carefully aspirated and 100 µL of DMSO were added to solubilize the formazan crystals. The absorbance of purple formazan was registered at 570 nm using a multiwell plate reader (Multiskan FC, Thermo Scientific). The cell viability was expressed as percentage values with respect to control cells, and the data are given as the mean value ± SD (standard deviation) of three independent experiments. Dose–response curves and the corresponding IC_50_ values were determined through nonlinear regression (curve fit), calculated with the GraphPad Prism 8.0.0 software [56].

#### Trypan blue test

A375 and A549 cells were seeded in a 6-well plate (2 × 10^5^ cells/well). After 24 h, the A375 and A549 cells were treated with 1.15 µM and 2.11 µM of compound **1**, that are the concentrations corresponding to the respective IC_50_ values. After 1 h of treatment, one plate was irradiated for 1 h with 700–400 nm light, whereas the other one not (control). After 2 days, non-adherent cells were collected with adherent cells after their trypsinization in each condition. The cell suspension was subsequently mixed with Trypan Blue 0.4% [1:1], and the percentage of dying (blue staining inside) and alive cells was determined and compared with their respective irradiated and non-irradiated non-treated cells.

### Confocal microscopy

A549 cells were cultured (3 × 10^4^ cells/well) in an 8-well sterile µ-Slide (Ibidi, Gräfelfing, Germany). The next day, the cells were treated with compound **1** (at both 2 and 20 µM) or with compound **2** (at 20 µM) for 1 h. Afterwards, the cells were irradiated (pre-irradiated) or not (dark conditions) with 700–400 nm light for 1 additional hour. In some experiments, the cells under dark conditions were irradiated for 5 min with the 488 nm fluorescent laser from a confocal microscope. To localize the drug inside the cells, it was excited with 405 nm laser and the emission between 594 and 754 nm was recorded. The differential interference contrast (DIC) image was used to characterize the different cellular structures. Images were taken using a Carl Zeiss LSM 880 spectral confocal laser scanning microscope (Carl Zeiss Microscopy GmbH, Jena, Germany) and analyzed with ZEN 2 blue edition software (Zeiss). Representative images from three independent experiments are shown.

## Results and discussion

### Preparation of the ligand Bndpa and Ru compounds 1 and 2

*N*-benzyl-2,2′-dipyridylamine (Bndpa) was synthesized by straightforward reaction of benzyl bromide with 2,2′-dipyridylamine in DMSO under basic conditions (KOH as base), following a reported procedure [47].

The complex precursor [Ru(phen)_2_Cl_2_] was prepared by reaction of RuCl_3_ with phen in DMF, in the presence of a reducing agent, namely hydroquinone, and an excess of LiCl (to minimize the formation of stable [Ru(phen)_3_]Cl_2_), following a procedure described for the synthesis of [Ru(biq)_2_Cl_2_] (biq = 2,2′-biquinoline) [48].

Complexes **1** and **2** were synthesized by reaction between equimolar amounts of [Ru(phen)_2_Cl_2_] and the corresponding ligand in refluxing methanol for 2 days under dinitrogen atmosphere. Both complexes **1** and **2** were isolated by precipitation upon addition of NH_4_PF_6_.

### Crystal structure of Ru compound 2

**Fig. 1:**
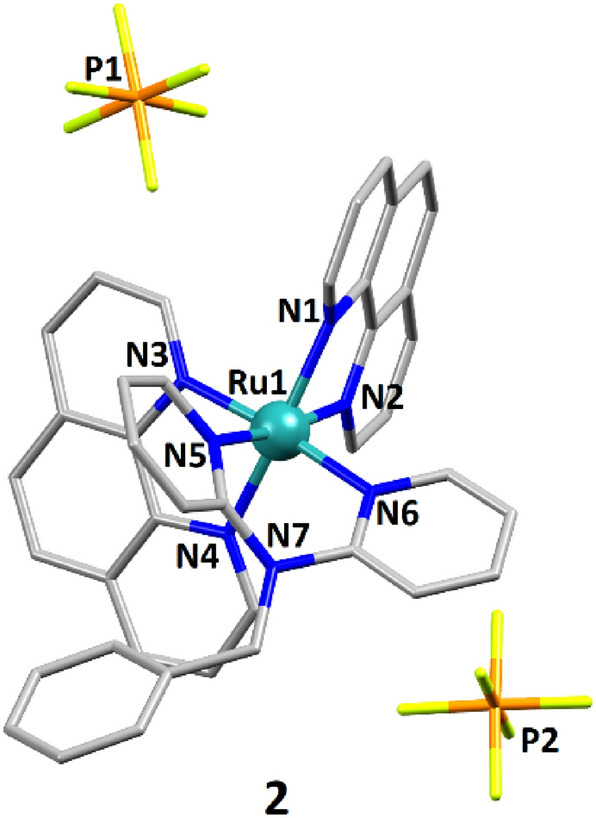
Representation of the crystal structure of **2**. The atoms coordinated to the metal centre, the metal ion, and the phosphorus atoms (PF_6_^−^ anions) are labelled. Hydrogen atoms and lattice solvent molecules are omitted for clarity

Single crystals of **2**, suitable for X-ray diffraction analysis, were obtained by slow diffusion of diethyl ether into a solution of the complex in acetonitrile. Complex **2** crystallizes in the monoclinic space groups *P*2_1_/c; the crystal structure of **2** is shown in Fig. 1. Crystallographic and refinement parameters are summarized in Table S2, and selected bond lengths and angles are listed in Table S3. The solid-state structure of **2** is subsequently compared to the reported one for **1**, which is depicted in Fig. S19A [49].

The coordination bonds of the octahedral RuN_6_ core span from 2.056(3) to 2.088(3) Å for **1** and from 2.059(3) to 2.088(3) for **2**. The angles vary from 79.9(1) to 97.0(1)° for **1** and from 79.6(1) to 97.9(1)° for **2** (Table S3); the same bonds and angles are in the range 2.056(4)–2.068(4) Å and 79.5(2)–99.4(2)°, respectively, for the reference compound [Ru(phen)_3_](PF_6_)_2_ (Fig. S19B) [57]. Thus, the geometries of both **1** and **2** cations are slightly *less distorted* than that of the “symmetrical” [Ru(phen)_3_]^2+^ cation; nevertheless, the range in bond distances for **1** and **2** is comparable to that of the reference [Ru(phen)_3_]^2+^ complex. The bite angles for the phen ligands are 79.9 and 80.1° for **1**, 79.6 and 79.8° for **2** and 79.5, 79.6 and 80.4° for [Ru(phen)_3_]^2+^. The phen bite angles are therefore comparable for the three compounds. The bite angle for the dpa complex is 86.9 and 85.5° for the complex with Bndpa, which are values close to those found for the complex [Ru(phen)(dpa)_2_], namely 86.9 and 88.5° [58]. It can also be pointed out that the dpa unit is more planar in **1** than in **2**, as reflected by the respective pyridine–N7–pyridine angles of 127.7 and 118.2° (Fig. S20A); thus, the angles between the planes containing the pyridine rings are of 37.2° and 51.8° for **1** and **2**, respectively (Fig. S20B). From these data, the steric hindrance of the benzyl group in Bndpa appears to affect the way in which the dpa unit is coordinated to the metal centre. To further analyse in detail the coordination environment of **1** and **2**, and compare it to that of the reference compound [Ru(phen)_3_](PF_6_)_2_, a series of structural parameters have been examined (Table 1). First, the average bond distance < Ru–N > (Å) is higher for **1** and **2** than that of [Ru(phen)_3_](PF_6_)_2_; the 2,2′-dipyridylamine ligands induce a slight expansion of the octahedron. In that context, the length distortion parameters *ζ *[59] for the three compounds indicate that the major deformation of the octahedron occurs for **1** ($\zeta$ values; Table 1). This is further confirmed by the angular distortion parameter Σ [59], where the furthest value from 90° is observed for **1** (Σ values; Table 1). Similarly, the mean quadratic elongation parameters λ (Table 1) also suggest that compound **1** exhibits the less regularized octahedral geometry. Finally, the bond angle variance σ^2^ is comparable for **1** and **2**, but significantly lower than that of [Ru(phen)_3_](PF_6_)_2_, which has the lowest distorted octahedral geometry.

**Table 1:** Structural parameters, namely the average bond distance, length distortion $\zeta$, angular distortion Σ, the mean quadratic elongation *λ* and the bond angle variance σ^2^, which characterize the octahedral distortion

	**1**	**2**	[Ru(phen)_3_](PF_6_)_2_
Average bond distance <Ru–N> (Å)	2.072	2.076	2.063
$\zeta ={\sum }_{i=1}^{6}\left|\left(Ru-{N}_{i}\right)-\langle Ru-N\rangle \right|$ [59]	0.0760	0.0460	0.0190
$\Sigma = {\sum }_{i=1}^{12}\left|90-{\phi }_{i}\right|$ [59]	58.79	60.40	68.74
$\lambda =\frac{1}{6}{\sum }_{i=1}^{6}{\left[\frac{({d}_{n}-\langle d\rangle )}{\langle d\rangle }\right]}^{2}$ [60]	4.1 × 10^−5^	2.0 × 10^−5^	3.7 × 10^−6^
${\sigma }^{2}=\frac{1}{11}{\sum }_{i=1}^{12}{({\theta }_{n}-90)}^{2}$ [60]	36.6	37.2	48.8

### UV–Vis and fluorescence spectroscopy

Electronic absorption spectra of 50 µM solutions of **1** and **2** in water were recorded at room temperature (Fig. S21).

Both complexes show an absorption maximum at 414 nm, which is attributed to Ru(t_2g_)-Ligand(π*) transitions (MLCT transitions) [41, 58, 61]. The absorption maxima observed in the 455–460 nm region are ascribed to n_dpa_-π*_phen_ and n_Bndpa_-π*_phen_ interligand transitions [62]. Finally, the π–π* ligand transitions are found at 223 nm and 264 nm [61, 63]. It is interesting to note that the spectra of both compounds are almost identical, indicating that the benzylation of the ligand dpa, *i.e.* Bndpa, does not alter the energy of the MLCT band significantly.

The emission spectra of **1** and **2** were recorded at room temperature in acetonitrile and in water, using 5 µM solutions and an excitation wavelength of 414 nm (Fig. S22). The emission maxima at 622 nm (complex **1**) and 607 nm (complex **2**) agree with reported values for ^3^MLCT excited state to ground state transitions of ruthenium(II) polypyridyl complexes [62, 63].

### Behaviour of 1 and 2 upon irradiation in solution

**Fig. 2:**
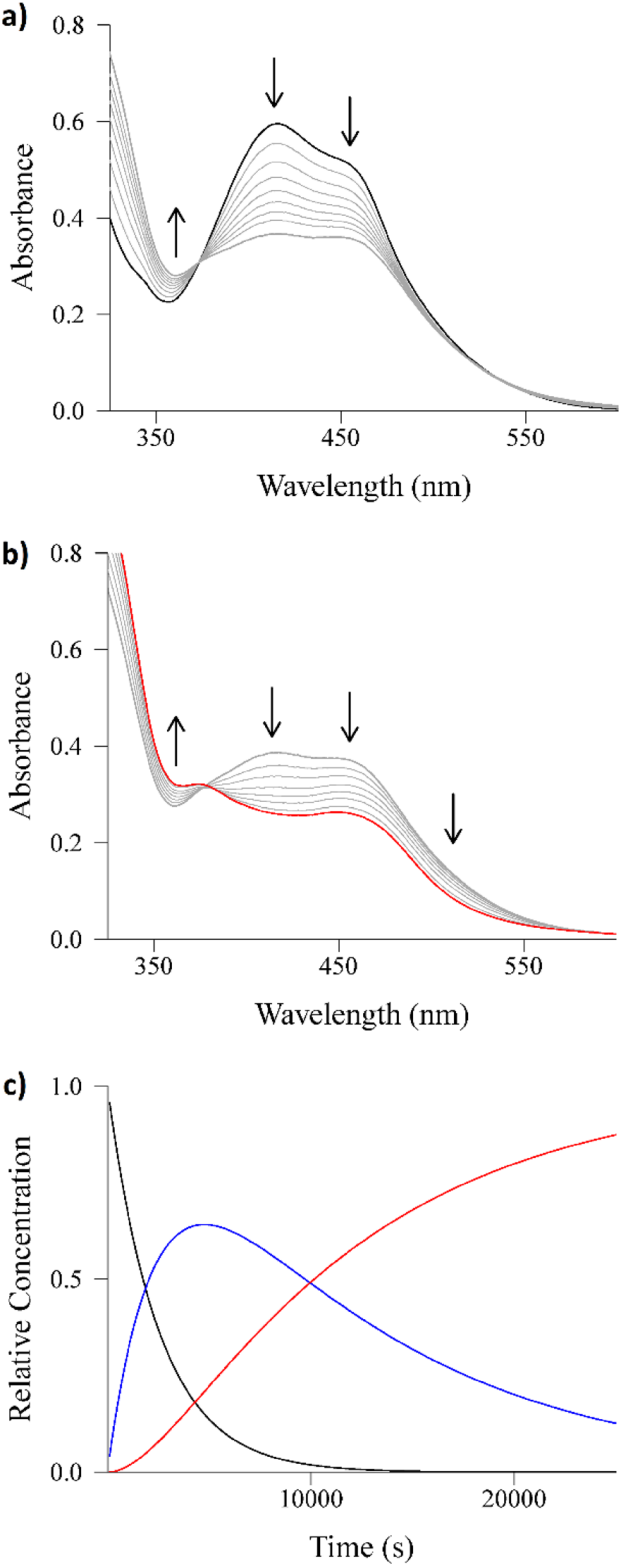
UV/Vis spectra of a 50 µM aqueous solution of **1** irradiated with 1050–430 nm light over time. **a** Step 1 (t_1/2_ = 2000s). **b** Step 2 (t_1/2_ = 5800 s). **c** Relative concentrations of initial complex (black), intermediate species resulting from step 1 (blue) and the final complex (red) after step 2

Mass spectra were recorded for **1** and **2** in acetonitrile, before and after 30 min of irradiation with 1050–430 nm light (Figs. S23 and S24). In both cases, the dpa-based ligands are lost after irradiation as free dpa and free Bndpa are observed in the corresponding spectra. Moreover, the {Ru(phen)_2_}^2+^ moiety can also be detected, further suggesting that dpa and Bndpa are ejected upon irradiation. For complex **1**, even the solvato-species [Ru(phen)_2_(H_2_O)_2_]^+^ and [Ru(phen)_2_(CH_3_CN)(H_2_O)]^+^ are present after irradiation (Fig. S23). These results prompted us to study the ejection of the dpa and Bndpa ligands from **1** and **2** by UV/Vis spectroscopy. The evolutions of the UV–Vis data for both complexes over time were thus obtained, in water and in acetonitrile solution, with continuous irradiation using 1050–430 nm light. The complete set of UV/Vis spectra obtained for **1** are shown in Figs. S25 and S26, and those for **2** in Figs. S27 and S28. Clearly a two-step process is observed for the two complexes in both solvents; Fig. 2a (step 1) and Fig. 2b (step 2) feature the occurrence of these two steps for complex **1**. The fitted relative concentrations of all the species involved are depicted in Fig. 2c. The corresponding UV/Vis data for complex **2** are shown in Fig. S29. The determined values of first-order rate constants (*k*_1_ and *k*_2_) are collected in Table 2.

**Table 2:** Values (in s^–1^) of the first-order rate constants (*k*_1_ and *k*_2_) determined from the changes in the spectra of solutions of compounds **1** and **2** over time upon 1050–430 nm irradiation [complex] = 50 µM, *T* = 25 °C

Solvent	Complex **1**		Complex **2**	
	*k*_1_ (s^–1^)	*k*_2_ (s^–1^)	*k*_1_ (s^–1^)	*k*_2_ (s^–1^)
Water	3.5 × 10^–4^	1.2 × 10^–4^	4.3 × 10^–4^	9.4 × 10^–5^
Acetonitrile	7.7 × 10^–2^	3.5 × 10^–4^	5.0 × 10^–2^	8.0 × 10^–4^

**Scheme 1:**
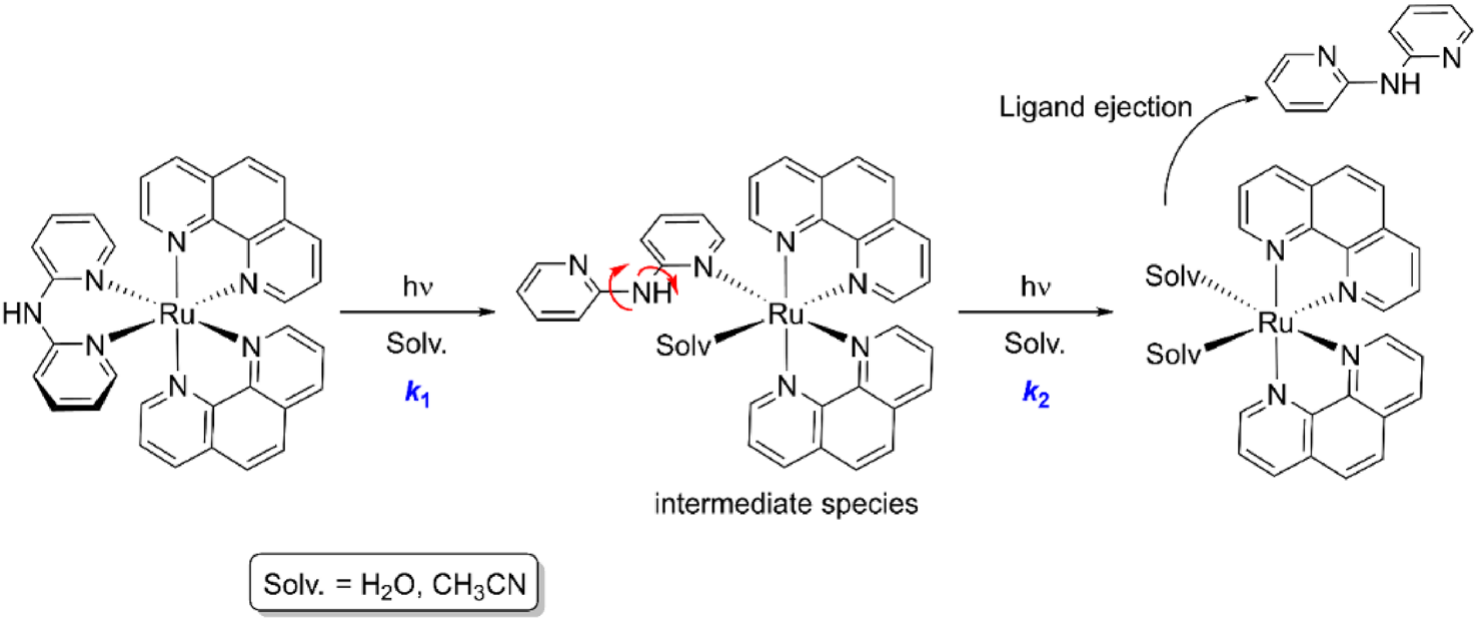
Proposed two-step photochemical process (characterized by ***k***_**1**_ and ***k***_**2**_) ultimately leading to the expulsion of the dpa ligand, which is replaced by solvent molecules (viz*.*, water or acetonitrile molecules in the present study). The red arrows illustrate the free rotation of the pyridine rings around the pyridine–NH bonds

The *k*_1_ and *k*_2_ values are comparable for the two complexes in a given solvent, but for the two species the first step is much faster (*ca.* two orders of magnitude) in acetonitrile (Table 2). While for both complexes, the rate constant for the second step is about half of that for the first step in water, the difference increases dramatically when acetonitrile is used (Table 2). Clearly, the effect derived from the distinct bpa-based ligands in compounds **1** and **2** is reflected mainly on the first solvolysis process, which is expected to produce a monohapto bidentated ligand (Scheme 1); from this point, the second step does not show these large differences. Such monohapto coordination of bidentate ligands resulting from a similar stepwise ligand-solvent exchange has been reported for [Ru(bpy)_2_(L_S_)]^2+^ units (L_S_ = 1,3-bis(methylthio)-2-propanol, 1,3-bis(methylthio)-2-methoxypropane, 1,3-bis(methylthio)-2-(carboxymethoxy)propane, 3,3′-dimethyl-2,2′-bipyridine) [64, 65]. Interestingly, the difference in the first step cannot be associated with Lewis basicity or size of the solved used, as water is a much better donor ligand than acetonitrile and not much larger. Thus, either differences in solvation of the dangling (ejected) pyridine substituent, or in solvent effects on the quantum yield, must be claimed as responsible for the facts observed. In any case, 75% completion of the photoreaction is attained after about 4 h at 25 °C.

Kinetic experiments were also carried out in aqueous solutions with 10 and 25 µM complex concentrations, aqueous 10% DMSO and cacodylate buffer with 10% DMSO. In all cases, the derived rate constants were found equivalent within the experimental error (Table S4). This indicates that none of the set up needed for the stock solutions of the complexes and DNA and in vitro cytotoxicity studies are relevant for the photoejection processes.

The dpa and Bndpa ligand ejection occurring upon irradiation of complexes **1** and **2** is not at all unprecedented. Several examples have already been reported [66, 67] for [Ru(phen)_2_L]X_2_ and [Ru(bpy)_2_L]X_2_ complexes (L = bidentate ligand) [39, 68], where the steric hindrance of the leaving ligand is commonly proposed as the origin of its photo-induced expulsion [35]. Nevertheless, the nature of the ligand is also a key factor, as observed for the complexes [Ru(phen)_2_(btz)](PF_6_)_2_, [Ru(bpy)_2_(btz)](PF_6_)_2_ and [Ru(4,4′-diMe-bpy)_2_(btz)](PF_6_)_2_ (with btz = 1,1′-dibenzyl-4,4′-bi-1,2,3-triazolyl), for which the leaving ligand (*i.e.* btz), is not sterically hindered [69]. In our case, the flexibility of the ligands may also play an important role in its expulsion by facilitating the occurrence of monohapto bidentate coordination; in fact, the flexibility of 2,2′-bipyridine, when compared to sterically hindered 2,9-diphenyl-1,10-phenanthroline (dpp), has been proposed to justify its expulsion upon irradiation of the complex [Ru(bpy)_2_(dpp)](PF_6_)_2_ [66].

**Fig. 3:**
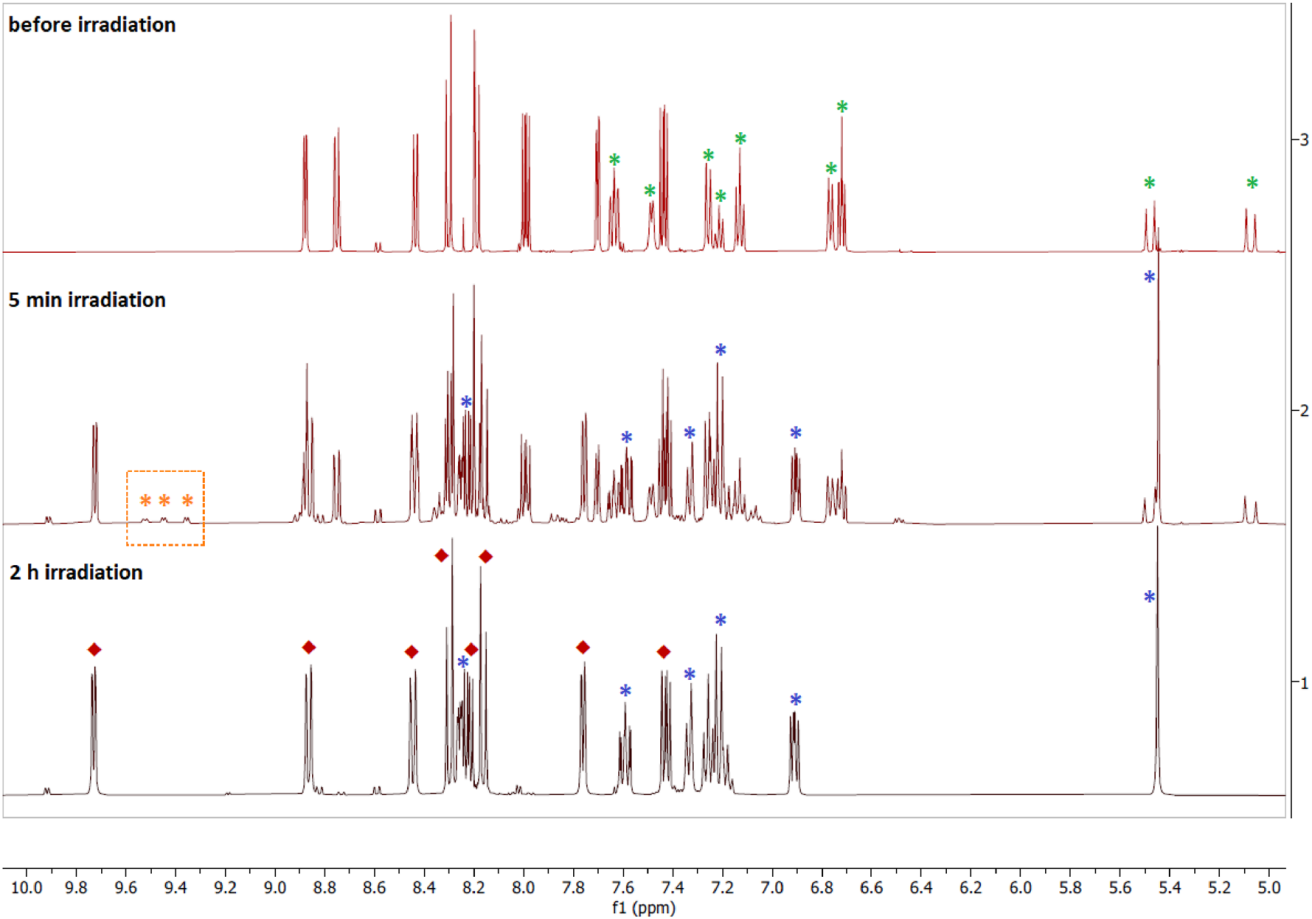
^1^H NMR spectra of complex **2** in CD_3_CN before irradiation (top), after 5 min of 1050–430 nm irradiation (middle) and 2 h of irradiation (bottom). The peaks marked with a green asterisk correspond to coordinated Bndpa, and those with a blue asterisk to free Bndpa. The peaks marked with a red diamond are ascribed to [Ru(phen)_2_(CH_3_CN)_2_]^2+^ [33]. The peaks marked with an orange asterisk (within the orange dotted box) are associated to the putative intermediate species

The identification of the product sequence obtained by irradiation of **2** in acetonitrile was conducted by means of ^1^H NMR spectroscopy (Fig. 3). As the irradiation time increases, the signals ascribed to the free Bndpa ligand grow (Fig. 3 in blue and Fig. S30), while those corresponding to coordinated Bndpa decrease (Fig. 3 in green and Fig. S30). Concomitantly, the signals for the phenanthroline protons shift to values that correspond to a [Ru(phen)_2_(L)_2_]^2+^ species [33], (*i.e.* [Ru(phen)_2_(CH_3_CN)_2_]^2+^, red in Fig. 3). It is to note that after 5 min of irradiation some transient signals are observed around 9.40–9.60 ppm (orange in Fig. 3 middle), which may be attributed to an intermediate species, most likely the putative [Ru(phen)_2_(Bndpa)(CH_3_CN)]^2+^ complex.

### Interaction of 1 and 2 with DNA

The photoejection of the bidentate, flexible ligand of **1** and **2** generate two (solvated) coordination sites that can favor the interaction of the “[Ru(phen)_2_]^2+^” species with DNA (solvent molecules replaced by nucleobases). This potential binding of irradiated **1** and **2** to DNA was investigated by gel electrophoresis, UV–Vis spectroscopy, fluorimetry (*i.e.*, Hoechst 33258 displacement assay) and circular dichroism.

#### Gel electrophoresis

The agarose gel electrophoresis was carried out as described in the “Experimental” section using 4 different types of samples, symbolized as P1–P4, which depend on the “light-exposure conditions” applied during their preparation. Hence, P1 correspond to the “*dark control*” where the complex and DNA were incubated for 30 min at 37 °C and kept in the dark at room temperature. P2 is a “*preirradiated sample*” for which the complex solution was irradiated for 30 min with 1050–430 nm light before the addition of DNA, and subsequent incubation at 37 °C for 30 min. P3 is an “*irradiated sample*” obtained by incubation of a solution of the complex and DNA for 30 min at 37 °C, which is then irradiated for 30 min with 1050–430 nm light. Finally, P4 corresponds to a “*preirradiated/irradiated sample*” prepared through irradiation for 30 min with 1050–430 nm light of a solution of the complex before addition of DNA. The resulting mixture is then incubated for 30 min at 37 °C and subsequently irradiated with 1050–430 nm light for 30 min.

**Fig. 4:**
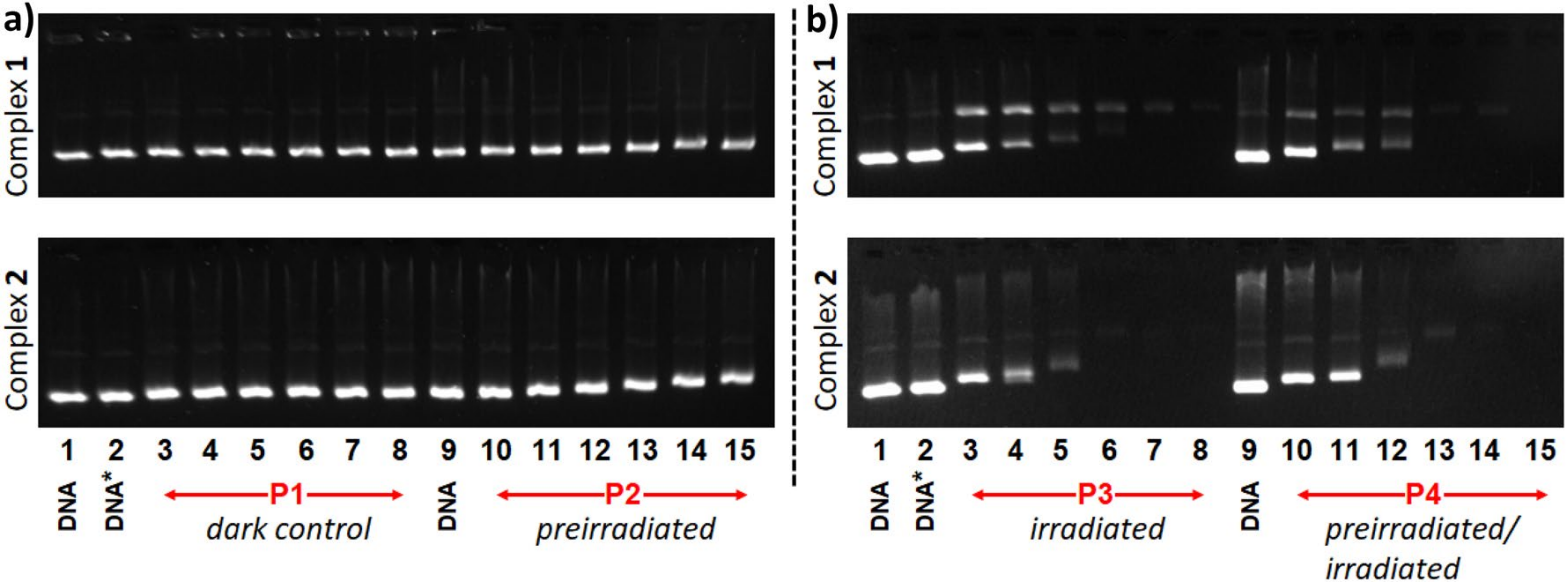
Agarose gel electrophoresis images of pBR322 plasmid DNA incubated with **1** and **2** using various complex concentrations (3.13 µM, 6.25 µM, 12.5 µM, 25 µM and 50 µM) and light-exposure conditions (**P1**–**P4** conditions; see main text for details). In all cases, lanes 1, 2 and 9 correspond to DNA control, DNA irradiated with 1050–430 nm light for 30 min (DNA*) and DNA control, respectively. Lanes 3–8 (**A**), 10–15 (**A**), 3–8 (**B**) and 10–15 (**B**) correspond to samples with increasing complex concentrations. **A** Conditions applied for complexes **1** and **2**: lanes 3–8, *dark control* (**P1**) samples without irradiation before or after incubation; lanes 10–15, *preirradiated* (**P2**) samples with irradiation before incubation. **B** Conditions applied for complexes **1** and **2**: lanes 3–8, *irradiated* (**P3**) samples with irradiation after incubation; lanes 10–15, *preirradiated/irradiated* (**P4**) samples with irradiation before and after incubation. [DNA] = 15 µM (in base)

The gel electrophoresis images obtained for **1** and **2** using the light conditions described above (P1–P4) and increasing concentrations of the complexes (from 1.56 to 50 µM) are displayed in Fig. 4. The *dark controls* (lanes 3–8 in Fig. 4a) do not show any alteration of the plasmid DNA (Form I) for both complexes. For the *preirradiated* samples (lanes 10–15 in Fig. 4a), a decrease of the DNA mobility is noticed when the concentration of the complex is increased, the effect being clearly more pronounced for **2** (Fig. 4a, bottom). Such a behaviour may indicate that the compounds induce an unwinding of the supercoiled structure of the biomolecule, a feature that is for instance observed upon DNA binding of cisplatin [70]. When **1** is irradiated after its previous incubation with DNA (P3 conditions), the formation of nicked plasmid (Form II) is observed together with the decrease of the electrophoretic mobility of Form I (lanes 3–8 in Fig. 4b, top image); the formation of Form II is indicative of photoinduced cleavage of the double-stranded molecule. Moreover, a diminution of the intensity of the SYBR™ Safe DNA stain is seen, which may arise from a reduced binding/intercalation of the dye as the result of the interaction of **1** with DNA. With **2**, DNA Form II is apparently not produced (lanes 3–8 in Fig. 4b, bottom image), the complex solely affecting the electrophoretic mobility of Form I. Applying the light-exposure conditions P4, viz. *preirradiated/irradiated* samples, the DNA is significantly more affected/damaged with both complexes (lanes 10–15 in Fig. 4b). The behaviours of **1** and **2** are comparable to those observed applying the conditions P3 (see above), but to a larger extent; indeed, lower concentrations of the compounds are required using P4 conditions to affect the biomolecule, compared with the P3 ones (Fig. 4b). It can be pointed out here that the free ligands dpa and Bndpa do not affect at all the DNA (Fig. S31); thus, the observed activities are due to the metal compounds.

**Fig. 5:**
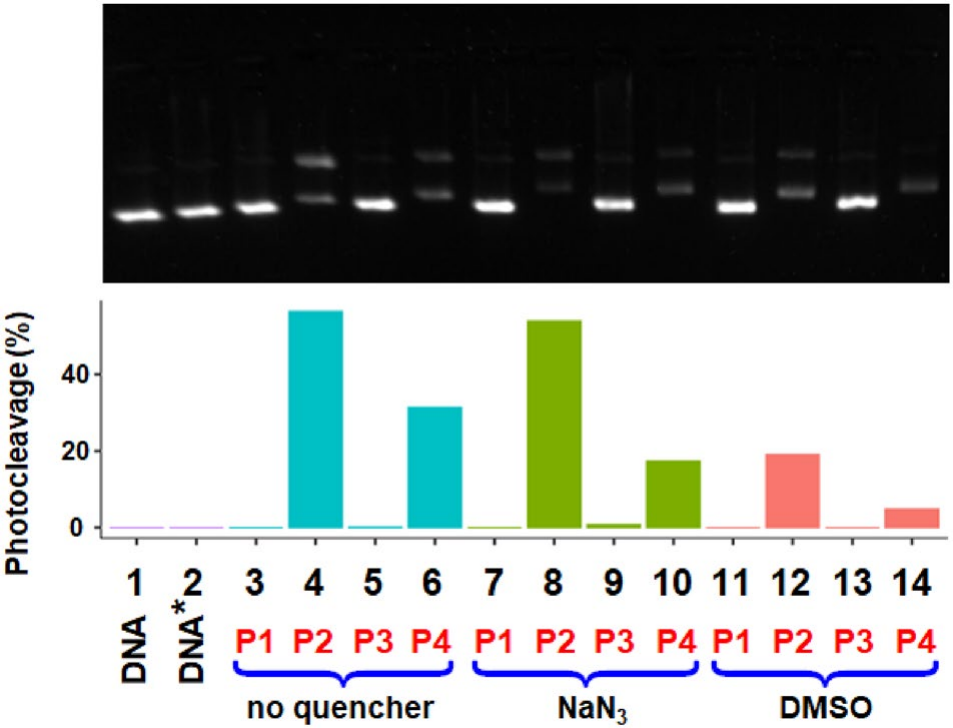
Top: agarose gel electrophoresis images of pBR322 plasmid DNA incubated with **1**, [complex] = 3.13 µM, using the light-exposure conditions P1–P4 (see main text for details), and without or with a ROS scavenger, viz. NaN_3_ (^1^O_2_ quencher) and DMSO (HO^·^ scavenger). Lane 1: DNA control, lane 2: DNA irradiated with 1050–430 nm light for 30 min, lanes 3–6: complex samples prepared applying conditions P1–P4 without scavenger present, lanes 7–8: complex samples prepared as previously but with NaN_3_ ([NaN_3_] = 5 mM), lanes 11–14: complex samples prepared applying conditions P1–P4 with 10% DMSO. [DNA] = 15 µM (in base). Bottom: percentages of photocleavage without scavengers (blue), in the presence of 5 mM NaN_3_ (green) and in the presence of 10% DMSO (red)

The photocleavage observed with **1** (generation of DNA Form II, Fig. 4b, top image) may originate from ROS generated upon irradiation of the complex. To examine whether ROS are produced when **1** is exposed to light, gel electrophoresis experiments were carried out with **1** in the presence of two scavengers, namely DMSO which is a hydroxyl-radical scavenger and sodium azide which quenches singlet oxygen. The corresponding electrophoretic results are shown in Fig. 5.

The two ROS scavengers only slightly reduce the effect of potential singlet oxygen or hydroxyl radicals (Fig. 5, top), DMSO being clearly more efficient than NaN_3_ (Fig. 5, bottom), hence suggesting that some HO^·^ are produced with **1**. Besides, the electrophoretic mobility of plasmid DNA appears to be slightly changed, suggesting that increased DNA binding occurs in the presence of the scavengers. Thus, the formation of DNA Form II does not solely occur through the reaction of Form I with ROS. The binding of complex **1** to DNA may be followed by a reaction that damages the biomolecule. For instance, plasmid DNA cleavage via direct oxidative damage at a guanine base or by a Type I sensitized reaction through electron transfer to the complex in its excited state was recently proposed for the peptide-containing complex [Ru(tap)_2_(bpyArCONH-ahx-VQRKQKLMP-CONH_2_]^6+^ (tap = 1,4,5,8-tetraazaphenanthrene) exhibiting an electrophoretic behaviour comparable to that of **1 **[71]. A related DNA degradation may therefore occur with irradiated **1**.

The effect of the irradiation time on a solution of DNA ([DNA]_b_ = 15 µM) and each complex ([complex] = 6.25 µM) was next examined. The corresponding electrophoresis image is depicted in Figure S32. In the case of **1**, the application of longer irradiation times gives rise to a progressive reduction of the mobility of DNA Form I and to an increase of the amount of DNA Form II (lanes 3–6 in Fig. S32), corroborating the dual effect of this complex, namely (i) binding leading to DNA unwinding and (ii) cleavage of the double-stranded molecule (see also Fig. 4b, top). For **2**, as already observed (Fig. 4b, bottom), the DNA-complex interaction affects the mobility of DNA Form I; this complex does not induce the generation of DNA Form II. It can be pointed out that when irradiation times of 15 and 30 min are applied, DNA is not detected (by the SYBR™ Safe dye), suggesting that strong DNA-complex interactions take place that release the dye or/and precipitated DNA-complex aggregates are formed, which are not detected in the gel.

**Fig. 6:**
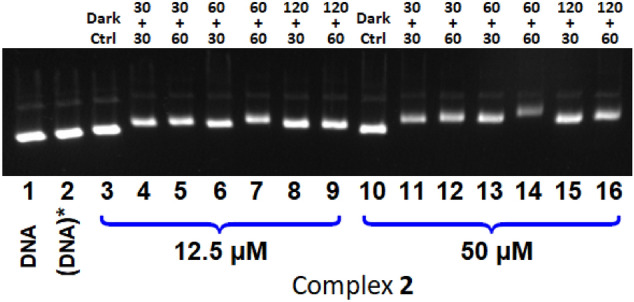
Agarose gel electrophoresis image of pBR322 plasmid DNA ([DNA]_bp_ = 15 µM) incubated with **2**, applying different pre-irradiation and incubation times and using two complex concentrations, namely 12.5 µM and 50 µM. Lane 1: DNA, lane 2: DNA with pre-irradiated buffer (DNA)*, lanes 3–9: [complex] = 12.5 µM, lanes 10–16: [complex] = 50 µM. Lanes 3 and 10, dark control. For lanes 4–9 and 11–16, the preirradiation and incubation times are mentioned above each lane in minutes, as preirradiation time + incubation time

This “DNA-mobility effect” induced by **2** was investigated further, by using different pre-irradiation and incubation times (viz. irradiation of **2** for a certain amount of time before incubation with DNA for a certain time) and two different complex concentrations, namely 12.5 and 50 µM. The electrophoresis image obtained is shown in Fig. 6.

First, the complex concentration has not a significant effect on the DNA electrophoretic mobility; only a slight diminution of the mobility is detected with the highest concentration (see lanes 7 and 14 or lanes 9 and 16). Increasing the pre-irradiation time maintaining constant the incubation time at 30 min (Fig. 6) does not alter the DNA mobility (see lanes 4, 6, 8 and lanes 11, 13, 15). Increasing the incubation time to 1 h (Fig. 6) slightly reduces the DNA mobility (see for example lanes 6 and 7 or 13 and 14); the longer incubation time obviously allows a higher DNA binding, therefore varying the electrophoretic mobility and the effect is logically more pronounced at the higher concentration. It can also be stressed that the lowest DNA mobility is observed when using a pre-irradiation time of 1 h and an incubation time of 1 h, with a complex concentration of 50 µM (Fig. 6, lane 14). When the pre-irradiation time of the previous sample is raised to 2 h (Fig. 6, lane 16), the DNA mobility is higher compared with that applying a pre-irradiation of 1 h (see lanes 14 and 16 in Fig. 6). This result may be explained by photodegradation of the complex upon longer light exposure, resulting in a reduction of the amount of the DNA-binding species; the DNA mobility is thus less affected. The same feature is noticed at lower complex concentration, *i.e.,* 12.5 µM (see lanes 7 and 9 in Fig. 6).

#### UV–Vis spectroscopy

UV–Vis DNA-binding studies with complexes **1** and **2** in cacodylate buffer were carried out as described in the “Experimental” section. First, UV–Vis spectra were recorded for a 25 µM solution of** 1** where increasing amounts of DNA were added ([DNA]_bp_/[complex] ratio varying from 0 to 1.06). The spectra obtained are shown in Figure S33a. Unsurprisingly, very little spectroscopic changes are observed, confirming the electrophoresis data using the P1 conditions, for which no alteration of the DNA mobility was noticed (see Fig. 4a top, lanes 3–8). When the complex solution is pre-irradiated for 1 h before the addition of DNA, the UV–Vis spectra obtained also exhibit minor variations, in agreement with the electrophoretic mobility observed applying the P2 conditions (see Fig. 4a top, lanes 10–15). For complex **2**, comparable results were obtained (Figs. S34a and S34b). A pre-irradiation time of 4 h was used as well with **2**; the corresponding UV–Vis spectra do not show any variations upon addition of DNA (Fig. S34c). However, it can be highlighted that the absorption bands are somewhat modified compared with those observed in Figures S34a and S34b; it appears that complex **2** experiences photodegradation upon long light exposure.

#### Hoechst 33258 displacement assay

Hoechst 33258 (H33258) is a minor groove binder whose fluorescence increases when it interacts with DNA. Therefore, its competitive displacement by an incoming molecule will result in fluorescence quenching that can be evaluated by determining the Stern–Volmer constant *K*_SV_, using the Stern–Volmer equation (see “Experimental” section for details). The fluorescence data (λ_exc_ = 352 nm; λ_em_ = 454 nm) achieved with complexes **1** and **2** are shown in Figures S35 and S36, respectively. Both under dark conditions and after irradiation of the compounds, quenching of H33258 fluorescence is noticed. Data fitting applying the Stern–Volmer equation produces non-linear plots (Figs. S34c and S36c); actually, the graphs have an exponential shape, which may be explained by a quenching of H33258 fluorescence by the complex that is simultaneously binding to DNA (through electrostatic interactions). In other words, H33258 may not be displaced but in fact is interacting with increasing amounts of the complexes (bound next to it) that quench its fluorescence (*e.g.*, through resonance energy transfer). For comparison purposes, apparent *K*_SV_ constants were calculated at the initial, linear portions of the graphs ([complex] < 9 µM). The values obtained are listed in Table 3.

**Table 3:** Apparent *K*_SV_ values (in M^–1^) determined at the initial linear portion of Stern–Volmer graphs obtained for complexes **1** and **2**, under dark conditions and with complex solutions that were previously irradiated. See Figs. S34c and S36c for the corresponding graphs

Complex	Dark conditions	Pre-irradiated complex
1	2.4 × 10^6^	1.4 × 10^7^
2	9.0 × 10^5^	2.3 × 10^7^

The Stern–Volmer data (Table 3) indicate that both complexes interact with DNA, as shown by the quenching of the fluorescence of H3325. The quenching effect is even more pronounced for the pre-irradiated complexes, which suggests a stronger binding of the “photoactivated” complexes. For **2**, this stronger interaction is apparently sufficient to start affecting the electrophoretic mobility of the biomolecule (see Fig. 4a, bottom; P2 conditions).

#### Circular dichroism

**Fig. 7:**
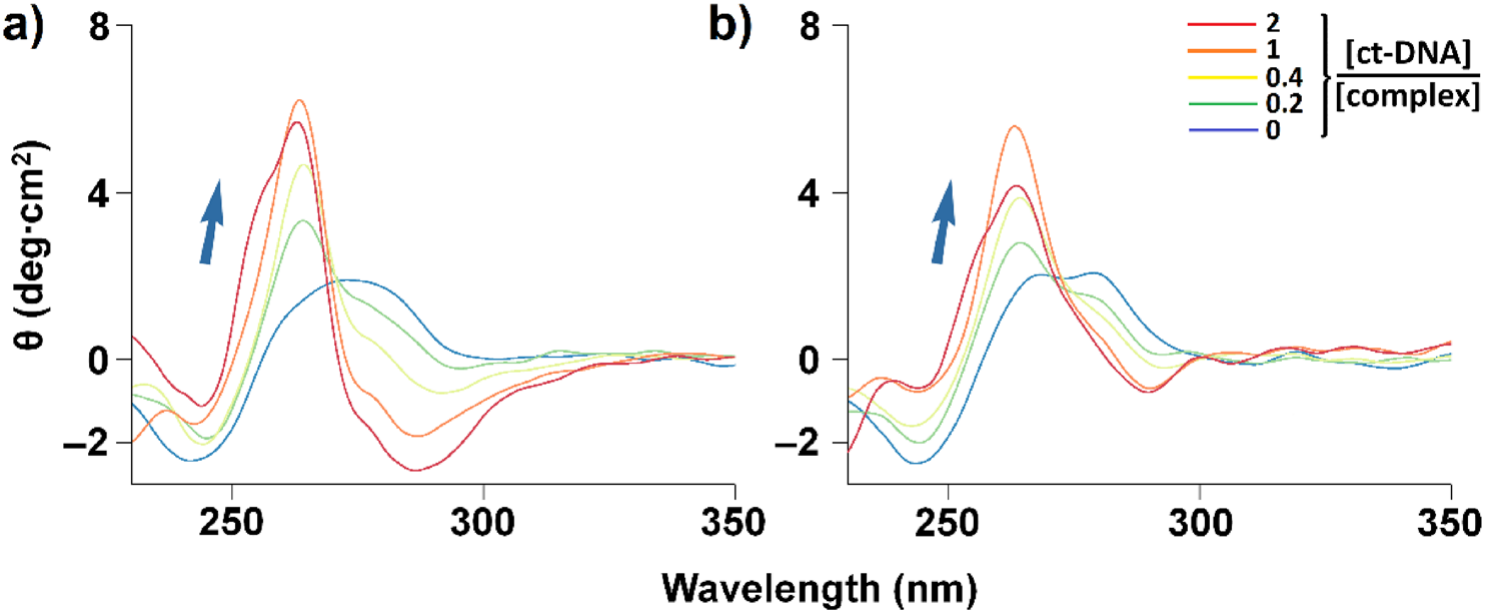
CD spectra of ct-DNA incubated with increasing amounts of complex **1**. [ct-DNA]/[complex] ratios = 0 (blue), 0.2 (green), 0.4 (yellow), 1 (orange) and 2 (red). [ct-DNA] = 50 µM. **a** CD spectra recorded under dark conditions; **b** CD spectra recorded using complex solutions that were pre-irradiated

Conformational changes of DNA (viz. secondary structure) can be investigated by circular dichroism (CD). CD spectra of *calf thymus* DNA (ct-DNA; 50 µM in base) incubated with increasing amounts of the complexes (from 5 to 50 µM) were recorded in the 220–350 nm range after 1 h incubation, under *dark* and *pre-irradiated* conditions. The corresponding spectra are shown in Figs. 7 and S37, for **1** and **2**, respectively. Significant modifications of the signals are observed upon addition of the complexes. The interaction of metal complexes with DNA commonly leads to slight peak shifts and a flattening of the typical CD spectrum [72]. In the present case, the interaction of both **1** and **2** strongly affects the bands observed in the initial spectra. For **1**, a broad negative signal at about 285 nm and a steeper positive around 265 nm grow upon addition of the complex (Fig. 7). It can be noticed that the development of the negative band is much less pronounced when pre-irradiated complex is used (Fig. 7b). The appearance of these signals suggest that a chiral molecule is present in solution; actually, the absorption values are comparable to those reported for the Δ isomer of bis-phenanthroline-containing ruthenium(II) complexes [33, 73, 74]. These features indicate that the Λ isomer of the complex preferentially interact with chiral biomolecule. Moreover, the fact that the signals of Δ isomer of **1** are almost solely visible suggest the interaction of the Λ isomer with B-DNA most likely generate complex-DNA aggregates that precipitate. For **2**, very similar features are observed (Fig. S37) indicating that the behaviours of both compounds are analogous. Incubation of the complexes with B-DNA for 24 h (instead of 1 h) does not give rise to significant differences (see Figs. S38a and 7a, and S38b and S37a for comparison), indicating that no thermal, DNA-binding equilibrium between the two enantiomers of the complexes takes place.

#### Cytotoxicity

The cytotoxic properties of complexes **1** and **2** were evaluated with two cell lines representing cancers that are easily accessible to light (*e.g.* skin cancer) [75] or potentially accessible to light through optical fibres (*e.g.* lung cancer) [76]. Hence, dose–response curves have been performed to determine half-maximum inhibitory concentrations (IC_50_ values) of complexes **1** and **2** incubated for 48 h with A375 cells (human melanoma) and A549 cells (human lung carcinoma), applying two conditions, namely the “dark conditions” and the “pre-irradiation conditions” (Fig. S38). As detailed in the “Experimental” section, **1** and **2** were first incubated with cells for 1 h at 37 °C to allow cell uptake. Afterwards, the “dark samples” were incubated for 1 h at room temperature protected from light and the “pre-irradiation samples” under irradiation with 700–400 nm light. Subsequent incubation for 48 h was performed before determination of the cell viability. It can be mentioned here that exposure of cells to light is known to reduce viability [77]. This light effect was indeed observed in the present study; therefore untreated but pre-irradiated cells (for 1 h) were used as a reference for the cell viability experiments to evaluate the cell death due to the irradiation and take it into account for the determination of the IC_50_ values.

The IC_50_ values obtained from the curves depicted in Fig. S38 are listed in Table 4. Both complexes are non-toxic if maintained in the dark (IC_50_’s > 100). Pre-irradiated **1** shows remarkable activities against both cell lines, namely 2.11 µM for A549 and 1.15 for A375 whereas pre-irradiated **2** remains inactive; hence, a simple benzylation of the dpa ligand, generating Bndpa, completely modifies the cytotoxic properties of the corresponding complex. A clear difference between **1** and **2** was already noticed by gel electrophoresis where the ability of **1** to cleave the DNA under light exposure was observed (Fig. 4b). Since the ligand is ejected upon irradiation, the cytotoxicity of the free ligands was also determined; it was found that dpa and Bndpa are not active under dark and pre-irradiation conditions (Table 4). Therefore, the behaviour of **1** is not due to the release of dpa but most likely to a photochemical process that affects the cells. Since the sole difference between **1** and **2** is the nature of the flexible ligand, viz. dpa or Bndpa, and that ligand ejection from both complexes generates identical ruthenium-containing species, one may reasonably assume that dpa is actually involved in the photocytotoxicity exhibited by **1** (namely, the complex with dpa still coordinated).

**Table 4:** Half-maximum inhibitory concentrations (IC_50_)^*a*^ in µM of complexes **1** and **2** and the free ligands dpa and Bndpa for A549 (lung adenocarcinoma) and A375 (melanoma) human cells, after incubation of 48 h at 37 °C

Complex	A549			A375		
	Dark^b^	Irradiated^c^	PI^d^	Dark^b^	Irradiated^c^	PI^d^
**1**	> 100	2.11 ± 0.58	> 50	> 100	1.15 ± 0.46	> 100
**2**	> 100	> 100	–	> 100	> 100	–
dpa	> 100	> 100	–	> 100	> 100	–
Bndpa	> 100	> 100	–	> 100	> 100	–

^a^The results are expressed as mean values ± SD out of three independent experiments (Fig. S39)

^b^Dark conditions: before the 48-h incubation, the compounds were incubated with cells for 1 h at 37 °C, to allow cell uptake, and another hour at RT protected from light

^c^Pre-irradiation conditions: before the 48-h incubation, the compounds were incubated with cells for 1 h at 37 °C, to allow cell uptake, and an additional hour at RT under irradiation (700–400 nm light)

^d^PI = phototoxic index obtained by IC_50_(dark)/IC_50_(irr)

To check whether the decrease in cell viability observed with pre-irradiated cells treated with **1** (MTT assay) was mainly caused by cell death, cells were stained after 48 h treatment with Trypan blue. The results revealed that **1** did not produce cell death in both cell lines under dark conditions (Fig. S40), hence corroborating the data achieved with the MTT assay. The percentage of alive cells lessened after pre-irradiation for the non-treated cells, although this was only significant with A375 cells (p < 0.001, data not shown). However, no increase in dead cells was observed compared to non-irradiated cells. These results suggest that irradiation for 1 h with 700–400 nm light reduces cell viability, for instance through induction of cell cycle arrest. Finally, a significant increase of the percentage of dead cells together with a reduction of the percentage of alive cells was observed in both pre-irradiated cell lines treated with **1**, compared to non-treated pre-irradiated cells (Fig. S40). These results therefore confirm that **1** is a potent cytotoxic agent when irradiated.

It can be pointed out that irradiated **1** is more active than cisplatin which shows IC_50_ values (after 72 h incubation) of 7.74 ± 1 for A549 cells [78] and 37 ± 2 for A375 cells [79]. The phototoxicity index (PI) is defined as the ratio between the compound’s activity in the dark and that under irradiation. For **1**, PIs of 50 and 100 were achieved for A549 and A375 cells, respectively. Comparable photocytotoxic properties have been described for ruthenium(II) polypyridyl complexes, but they sometimes show notable activities under dark conditions as well [35, 41, 80]. Various examples of PI values above 100 and even up to 500,000 have been reported [35, 36, 78, 81].

Finally, it can be mentioned here that photocytotoxic properties of [Ru(bpy)_2_(dpa)](PF_6_)_2_, namely the 2,2′-bipyridine analogous complex to **1**, have lately been reported with HL60 cells (acute promyelocytic leukemia) [82]. Hence, this complex is inactive in the dark while it exhibits an IC_50_ value of about 25 µM (PI of about 4 when irradiated for 1 min with 450 nm light). As in the present study, dpa was found to be inactive against this cell line. Taking into account that it has been shown that [Ru(bpy)_2_(OH_2_)_2_]^2+^ does not seem to be a cytotoxic species [27], one may consider that the anticancer behaviour of [Ru(bpy)_2_(dpa)](PF_6_)_2_ occurs during light irradiation, as for **1**.

#### Subcellular localization

**Fig. 8:**
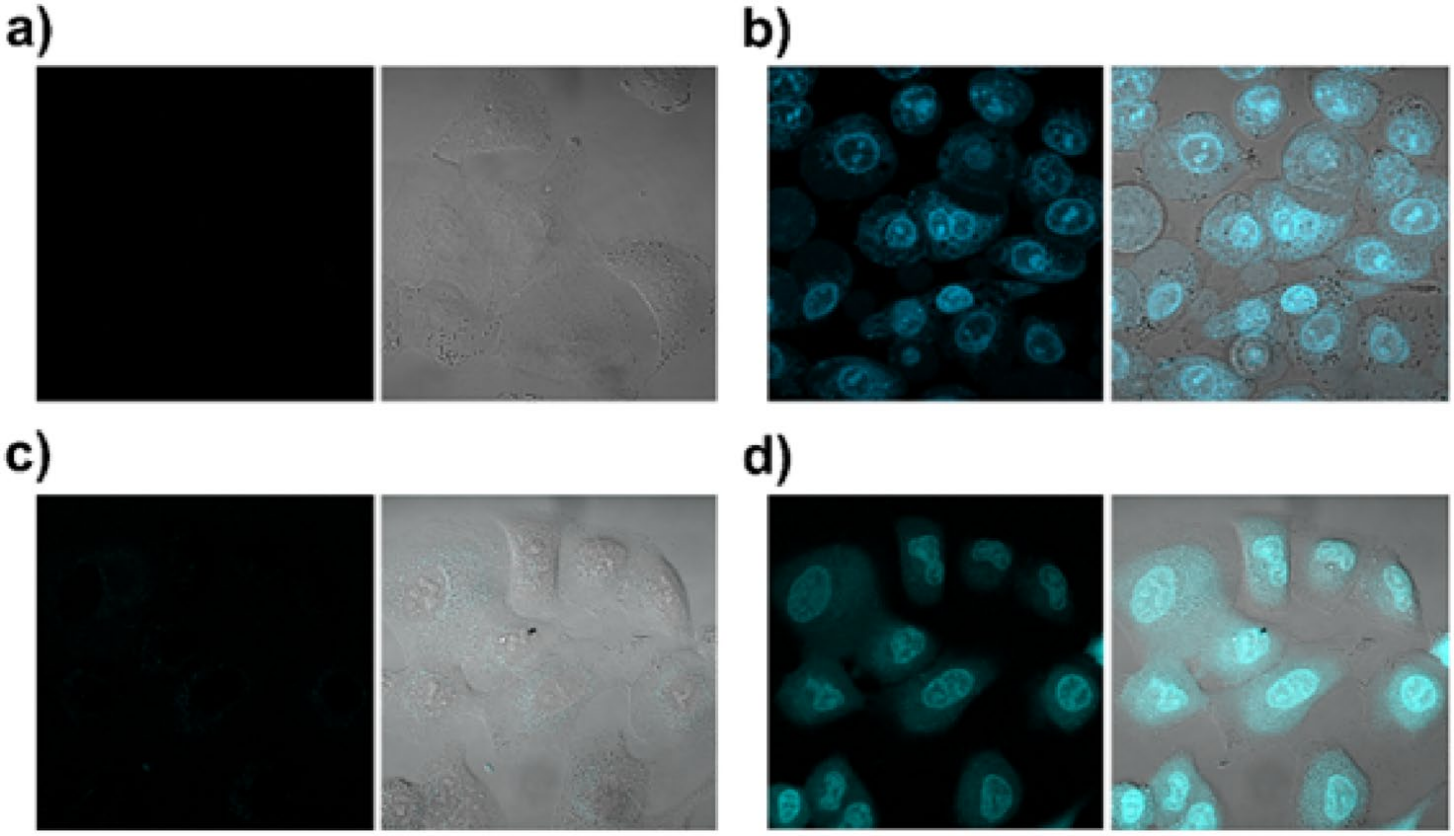
Localization of compound **1** inside A549 cells using confocal microscopy. Left images = drug emission; right images = merge of the drug emission with the DIC image contrast. **a** Non-treated cells pre-irradiated for 1 h with 700–400 nm light. **b** Cells treated with 20 µM of **1** for 1 h and subsequently irradiated with 700–400 nm light for additional 1 h. **c** Cells treated for 2 h with 20 µM **1** under dark conditions. **d** Cells treated for 2 h with 20 µM of **1** under dark conditions and subsequent irradiation for 5 min at 488 nm with the laser of the confocal microscope

To assess if the two compounds can bind DNA inside living cells, their subcellular localization was investigated by confocal microscopy using a [compound] of 20 µM. Compound **2** could not be observed due to its lack of fluorescence, even when irradiated. The weak fluorescence exhibited by compound **1** under the conditions applied was still enough to be detected, especially when it was pre-irradiated. Pre-irradiated (1 h with 700–400 nm light), non-treated cells did not show fluorescence (Fig. 8a). In contrast, pre-treated cells with compound **1** (for 1 h), preferentially accumulated inside the nucleus and mostly inside the heterochromatin when irradiated (Fig. 8b). Cells treated with **1** under dark conditions (hence exhibiting even lower fluorescence compared to pre-irradiated cells) showed the presence of **1** inside the cytosol and in some structures near the nucleus (Fig. 8c). Due to its low emission, it was not possible to use organelle trackers. Finally, the irradiation of cells pre-treated with **1** under dark conditions, with the 488 nm laser of a confocal microscope for 5 min, showed an increase of the fluorescence of the compound, which started to be localized inside the nucleus (Fig. 8d). It can be pointed out here that all microscopy experiments described above for a [compound] of 20 µM were also carried out with a [compound **1**] of 2.1 µM (viz., the IC_50_ value for **1** in A549 cells), and similar results were obtained but with significantly lower definition (data not shown). In summary, the microscopy data achieved indicate that **1** most likely binds DNA inside the cell nucleus after irradiation.

#### Lipophilicity

The lipophilicity of complexes **1** and **2** was determined using the shake-flask procedure, which was used to calculate their partition coefficients in an octan-1-ol (o)/water (w) system [83, 84]. Hence, the lipophilicity of the compounds can be expressed as the logarithm of the partition coefficients using these solvents (log *P*_o/w_), which can be estimated using Eq. (3):

3 $$\log P_{{\text{O}}/{\text{W}}} = {\text{log}}\left( {\frac{{{\text{Acs}}}}{{{\text{Afs}} - {\text{Acs}}}}} \right)$$

where A_fs_ is the absorption value corresponding to the MLCT band of the compound after partition in water saturated with octan-1-ol and A_cs_ is the absorption value of the MLCT band after subsequent partition in octan-1-ol saturated with water (see “Experimental” section for details). The corresponding UV–Vis spectra for **1** and **2** are shown in Figures S15 and S16, respectively. From these spectroscopic data (experiments carried out in triplicate), log *P*_o/w_ values could be obtained for both complexes (Table S1), namely 1.93 ± 0.02 for **1** and 2.04 ± 0.03 for **2**. These positive values characterize a lipophilic character, the slight variation in log *P*_o/w_ indicating a minor difference in hydrophobicity between them; therefore, their behaviour in aqueous solutions is expected to be comparable.

## Conclusions

It is widely accepted that PACT with Ru(II) polypyridyl complexes involves the photoinduced release of a ligand, producing cytotoxic species. The observed toxicity may be due to the free ligand, or the generation of (di-)aquated Ru(II) species that can, for instance, bind to DNA. Furthermore, it is also believed that the increase of steric bulk near the metal center favors the photodissociation of the ligand from the distorted octahedral complex.

Two ruthenium(II) complexes with simple, non-sterically hindered ligands have been prepared by standard procedures. Both heteroleptic complexes, viz. [Ru(phen)_2_(dpa)](PF_6_)_2_ (**1**) and [Ru(phen)_2_(Bndpa)](PF_6_)_2_ (**2**), show octahedral geometries that are more distorted than that of the parent homoleptic complex [Ru(phen)_3_](PF_6_)_2_, although the steric bulk is not significantly different for the three compounds. For both complexes, a very efficient dpa (**1**) or Bndpa (**2**) release is observed upon light irradiation; the full reaction involves a two-step process with respective half-life times of *ca.* 2000 and 6000 s, which are fairly equivalent for both species. Although complete photodissociation of the ligand is slightly slower for complex **2**, total ligand ejection is observed for **1** and **2** after several hours of irradiation, as already observed for other non-sterically hindered ligand complexes. DNA-binding studies (using various techniques) revealed distinct behaviours of the two complexes. While **2** exclusively affects its electrophoretic mobility, **1** also produces some damages, as evidenced by the formation of form II, *nicked circular*, of DNA. The cleavage mechanism of **1** does not seem to involve the major formation singlet oxygen nor any radical species; additional studies will be carried out with other similar model compounds to assess the mode of action of this family of complexes. Cytotoxic studies have also been conducted with rather striking results. Complex **1** showed the most interesting properties, since, while not active under dark conditions, it becomes significantly cytotoxic when irradiated for 1 h. Surprisingly, compound **2** (where the NH group of dpa has been benzylated) does not show any cytotoxicity, neither under dark nor irradiated conditions. Considering that the two starting materials (**1** and **2**), as well as their free ejected ligands (dpa and Bndpa), are not cytotoxic, and that both coordination compounds ultimately should generate the same solvated ruthenium(II) species ([Ru(phen)_2_(OH_2_)_2_]^2+^) upon irradiation-promoted ligand ejection, the activity exhibited by **1** has to arise from a species existing between the two structures, putatively the monohapto [Ru(phen)_2_(η^1^-dpa)(OH_2_)]^2+^ transient complex. As a whole, we should stress that while the full photoejection of one of the ligands normally represents a key step in the cytotoxicity mechanism of reported Ru(II) polypyridyl complexes, here it corresponds to an inactivation mechanism. The photocytotoxic behaviour of complexes analogous to **1** are currently under investigation, and a special focus is being set on the potential role played by the NH group of dpa-type ligands during the photochemical process.

## Supplementary Information

Below is the link to the electronic supplementary material.

Supplementary file 1 (PDF 3450 KB)

Supplementary file 2 (CIF 1407 KB)

Supplementary file 3 (PDF 114 KB)

## Data Availability

All these data can be found as Supporting Information (accessible online).
