# Supplementary material for: Steric hindrance, ligand ejection and associated photocytotoxic properties of ruthenium(II) polypyridyl complexes
Source: J Biol Inorg Chem. 2023 Apr 15;28(4):403–20. doi: 10.1007/s00775-023-01998-z (PMC10149480; doi:10.1007/s00775-023-01998-z)
Supplement: Supplementary file 1 — (PDF 3450 KB) [file 775_2023_1998_MOESM1_ESM.pdf]

# Steric hindrance, ligand ejection and associated photocytotoxic properties of ruthenium(II) polypyridyl complexes

Piedad Herrera-Ramírez[1♦] • Sarah Alina Berger[1♦] • Dana Josa[1] • David Aguilà[1,2] • Ana B. Caballero[1,2] • Pere Fontova[3,4] • Vanessa Soto-Cerrato[4,5] • Manuel Martínez[1,2✉] • Patrick Gamez[1,2,6✉]

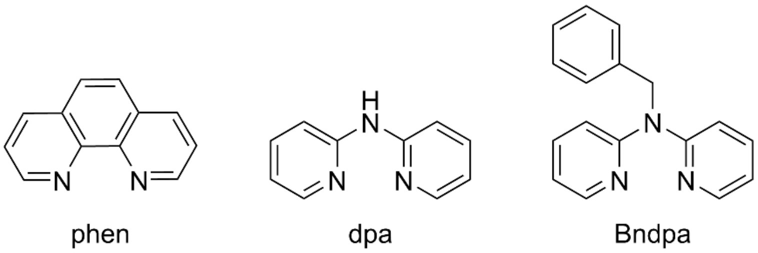


**Figure S1.** N-donor ligands used to prepare complexes **1** and **2**, namely 1,10-phenanthroline (phen), 2,2’-dipyridylamine (dpa) and *N*-benzyl-2,2’-dipyridylamine (Bndpa).

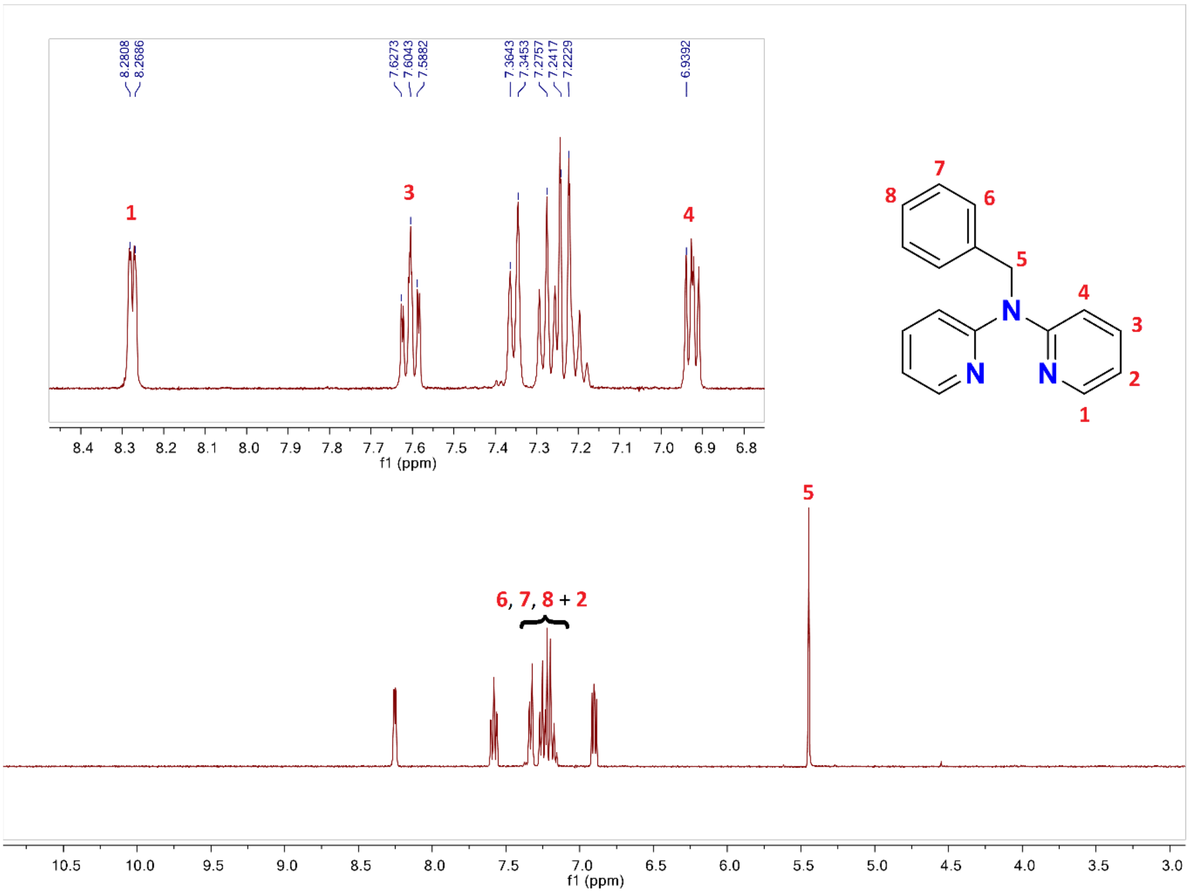


**Figure S2.** $^1$H NMR spectrum of ligand Bndpa.

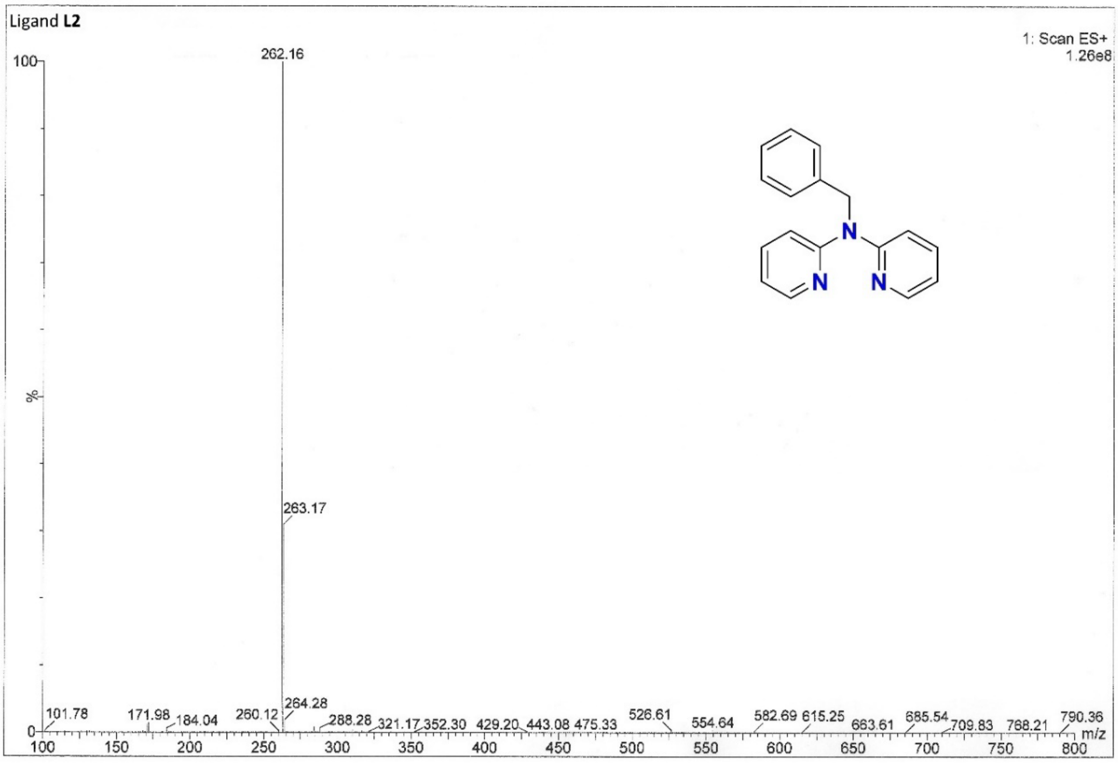


**Figure S3.** ESI-MS spectrum of ligand Bndpa. LRMS (ES+) m/z [M+H]$^+$ calcd. for $C_{17}H_{16}N_3$ 262.34.

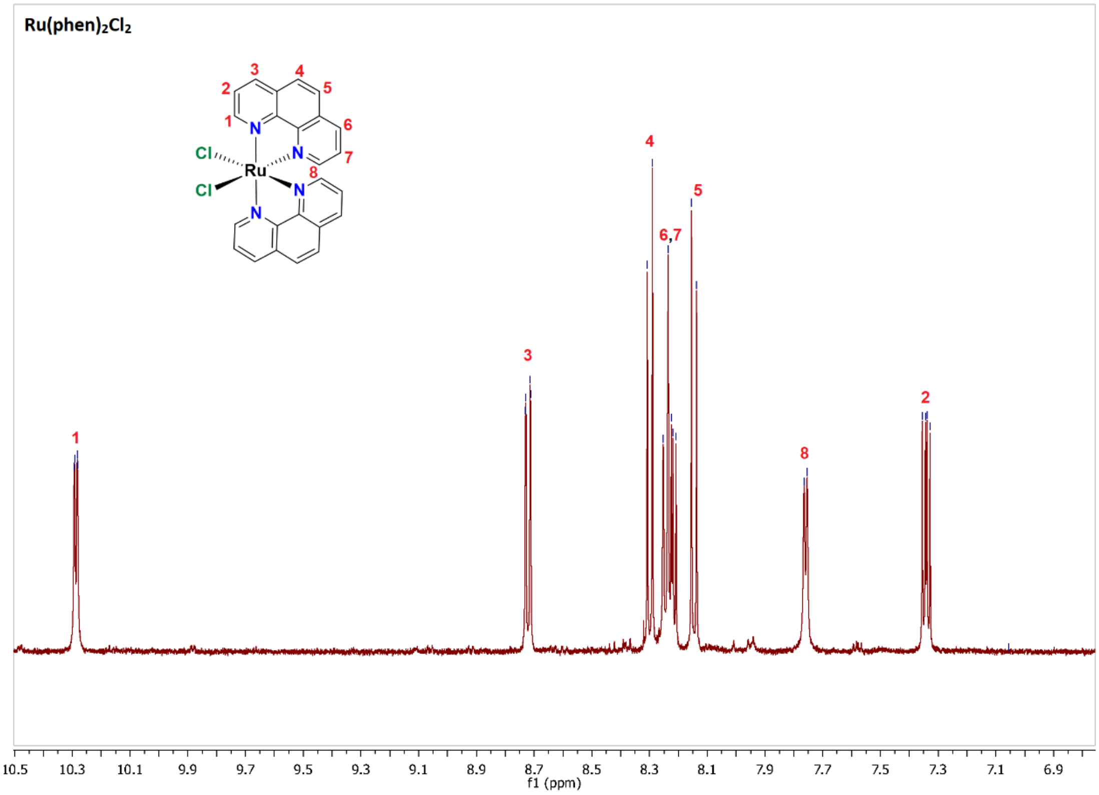


**Figure S4.** $^1$H NMR spectrum of **Ru(phen)$_2$Cl$_2$**.

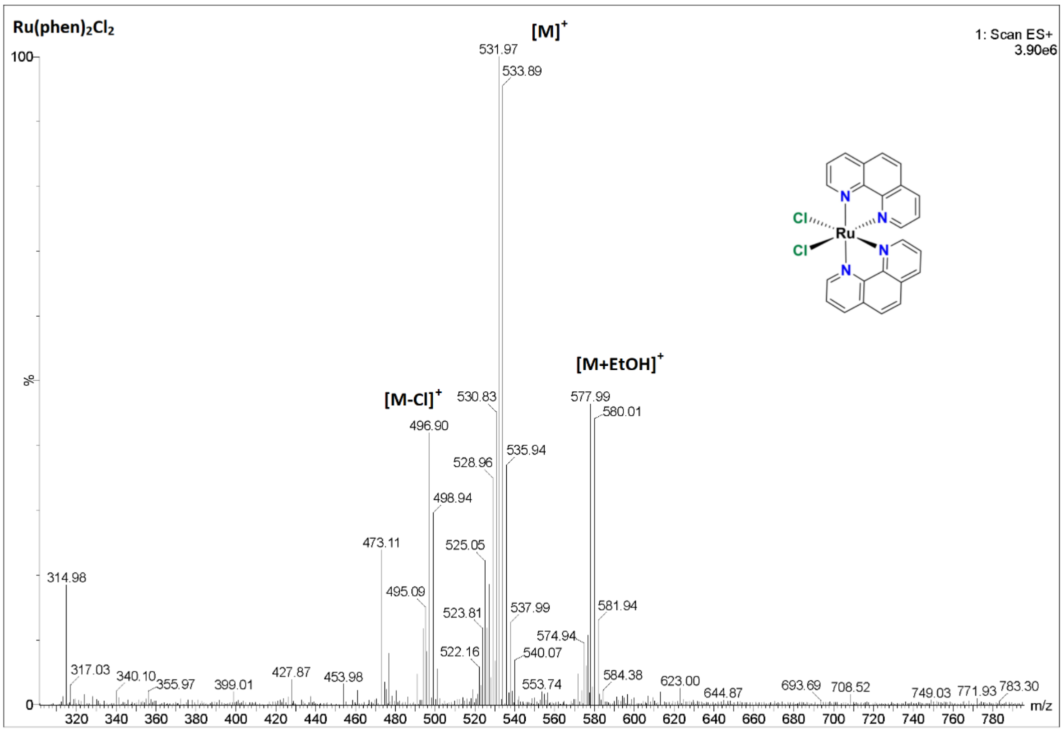


**Figure S5.** ESI-MS spectrum of ligand **Ru(phen)$_2$Cl$_2$**. LRMS (ES+) m/z $[M]^{2+}$ calcd. for $C_{24}H_{16}Cl_2N_4Ru$, 531.35; m/z $[M-Cl]^+$ calcd. for $C_{24}H_{16}ClN_4Ru$, 497.01; m/z $[M+EtOH]^+$ calcd for $C_{26}H_{22}Cl_2N_4ORu$, 578.02.

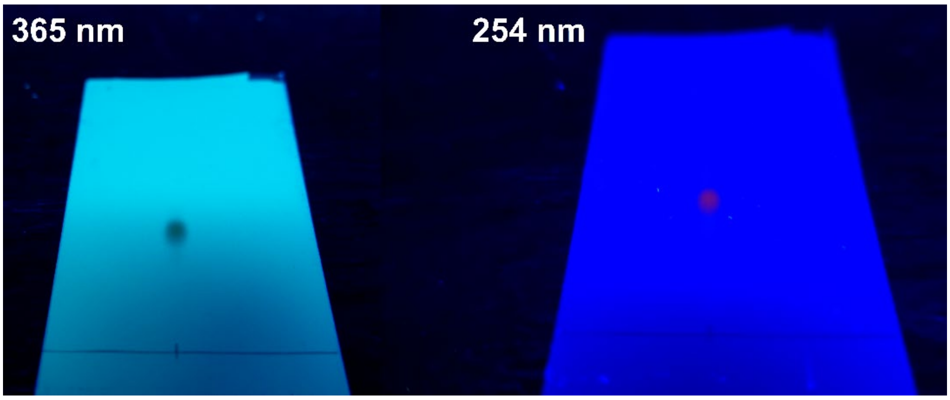


**Figure S6.** TLC run for compound **1** using $CH_3CN$ 9/0.1M $KNO_3$ 1, observed under UV light (365 and 254 nm).

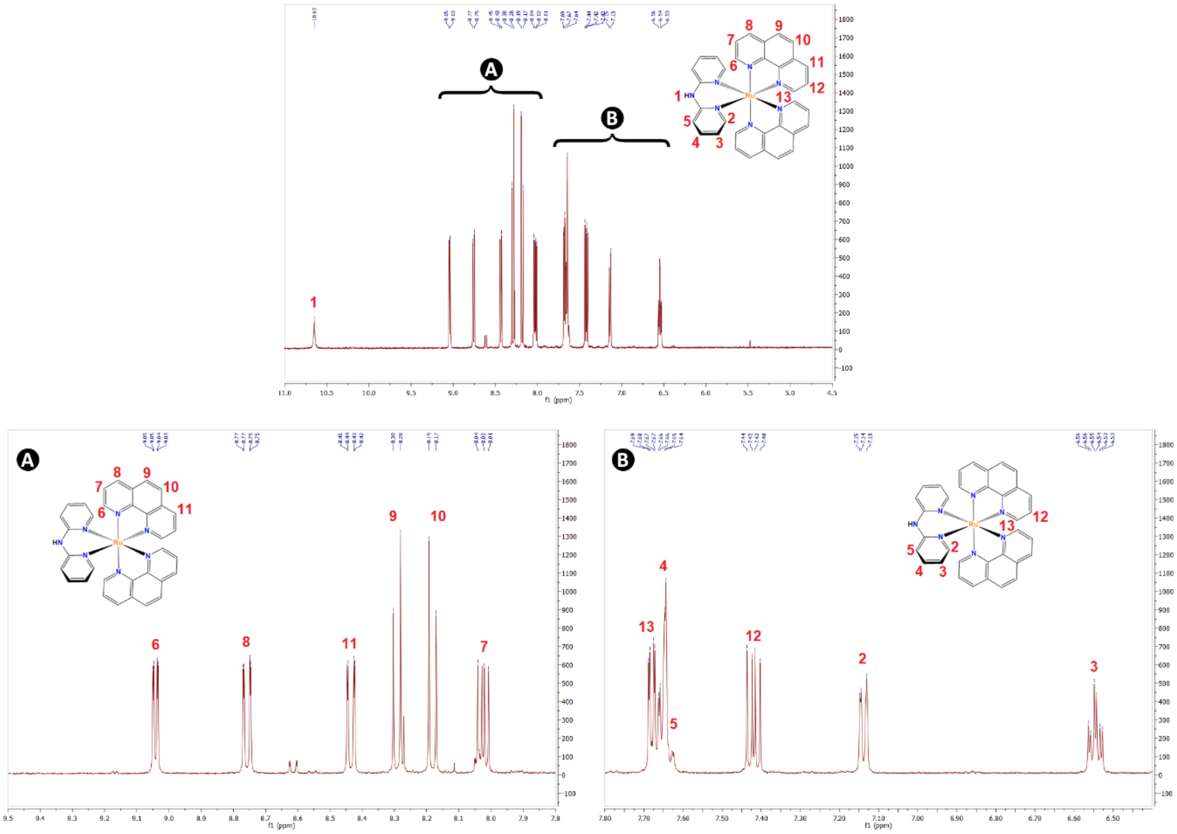


**Figure S7.** $^{1}H$ NMR spectrum of $[Ru(phen)_2(dpa)](PF_6)_2$ (**1**). Top: complete spectrum; bottom left: zoom **A** (between 9.5 and 7.8 ppm); bottom right: zoom **B** (between 7.8 and 6.4 ppm).

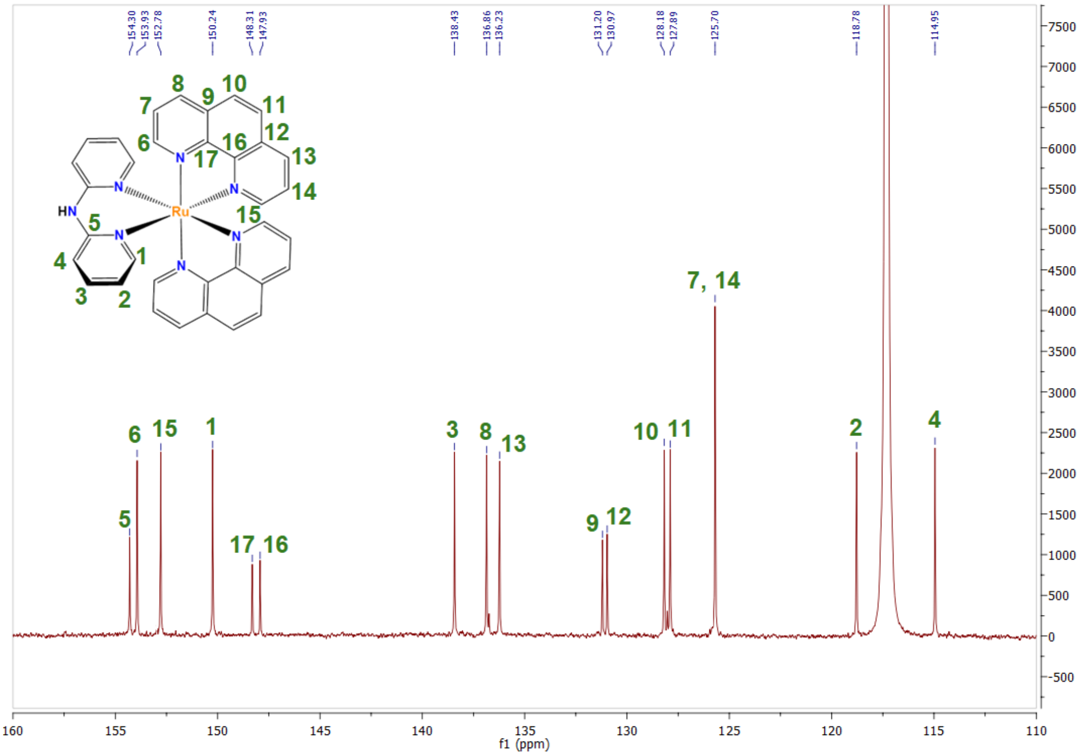


**Figure S8.** $^{13}C$ NMR spectrum of $[Ru(phen)_2(dpa)](PF_6)_2$ (**1**) in $CD_3CN$.

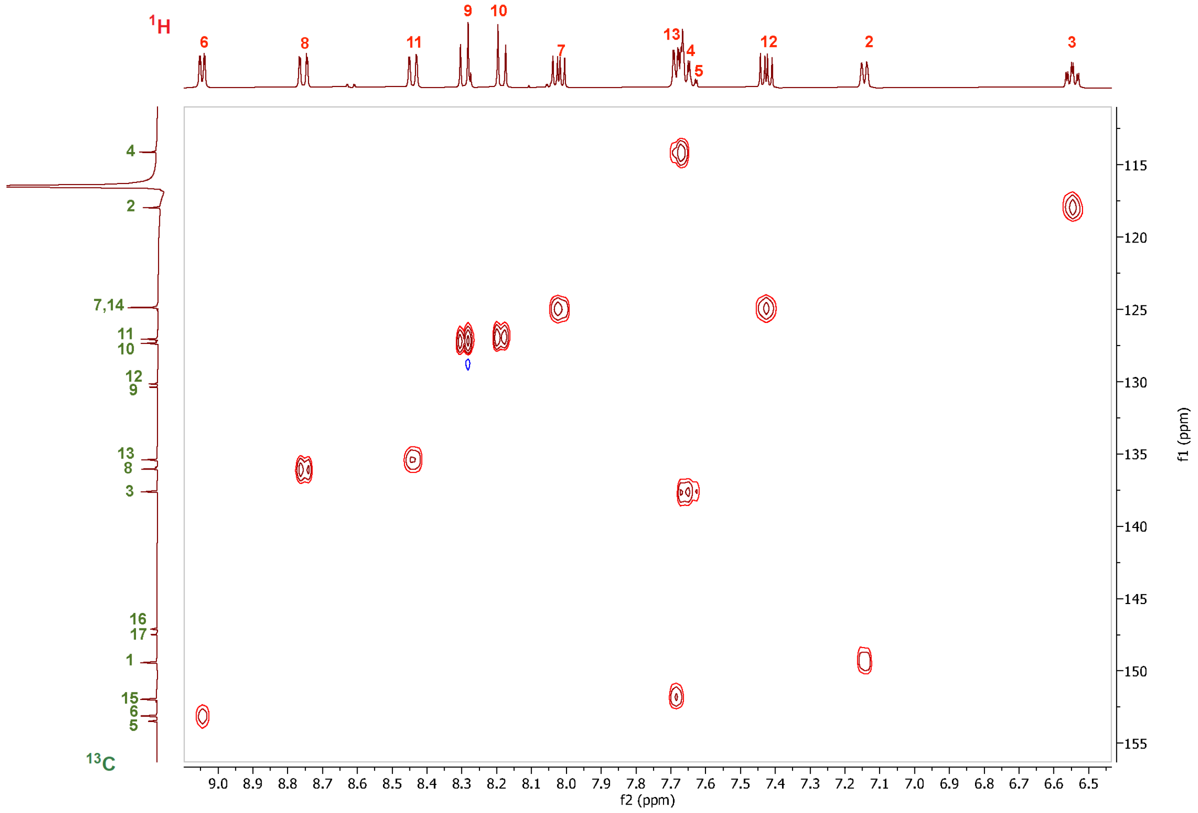


**Figure S9.** $^{1}H$-$^{13}C$ HSQC NMR spectrum of [Ru(phen)$_2$(dpa)](PF$_6$)$_2$ (**1**). The **H**- and **C**-atom numbering are shown in **Figures S6** and **S7**, respectively.

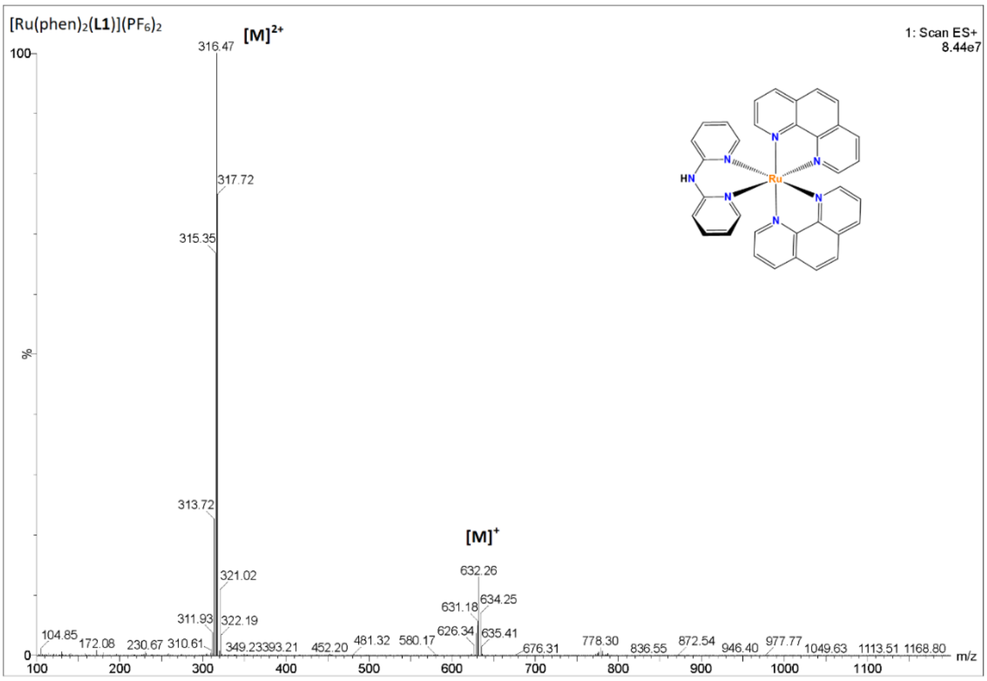


**Figure S10.** ESI-MS spectrum of ligand [Ru(phen)$_2$**L1**](PF$_6$)$_2$ (**1**). LRMS (ES+) m/z [M]$^{2+}$ calcd. for $C_{34}H_{25}N_7Ru$, 316.35; m/z [M]$^+$ calcd. for $C_{34}H_{25}N_7Ru$, 632.69.

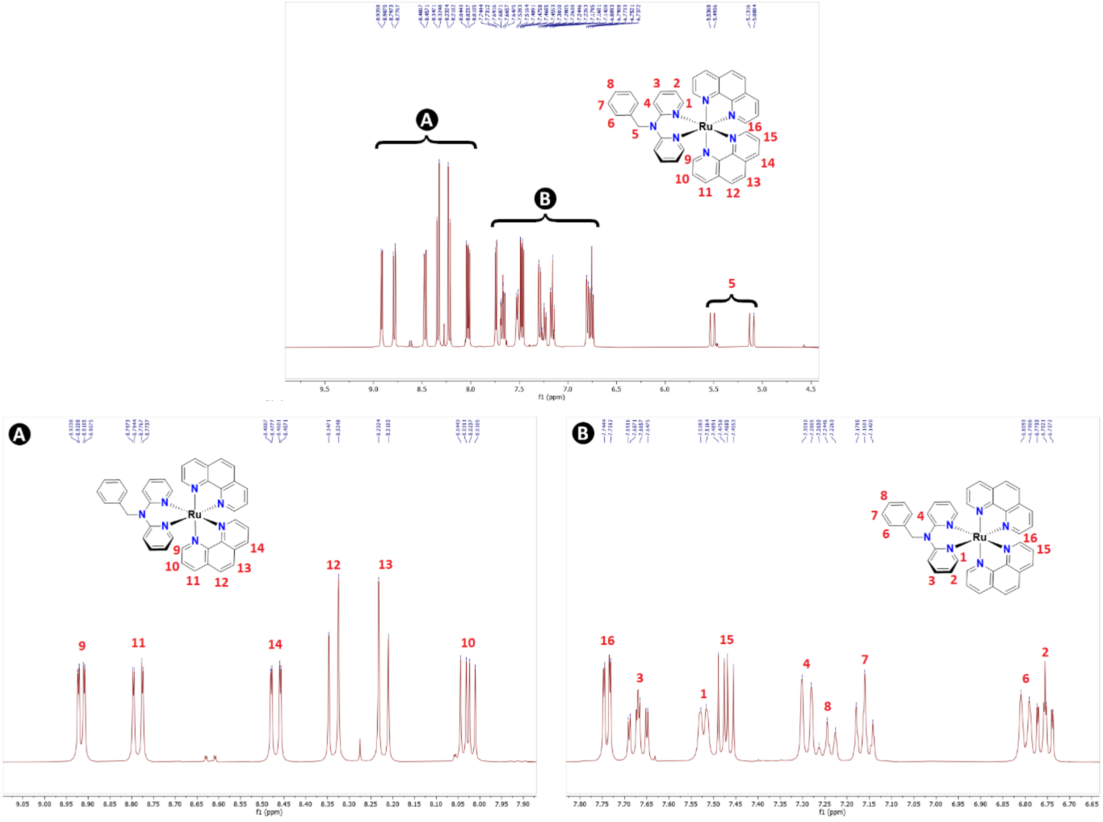


**Figure S11.** $^1$H NMR spectrum of [Ru(phen)$_2$**L2**](PF$_6$)$_2$ (**2**). Top: complete spectrum; bottom left: zoom **A** (between 9 and 8 ppm); bottom right: zoom **B** (between 7.80 and 6.65 ppm).

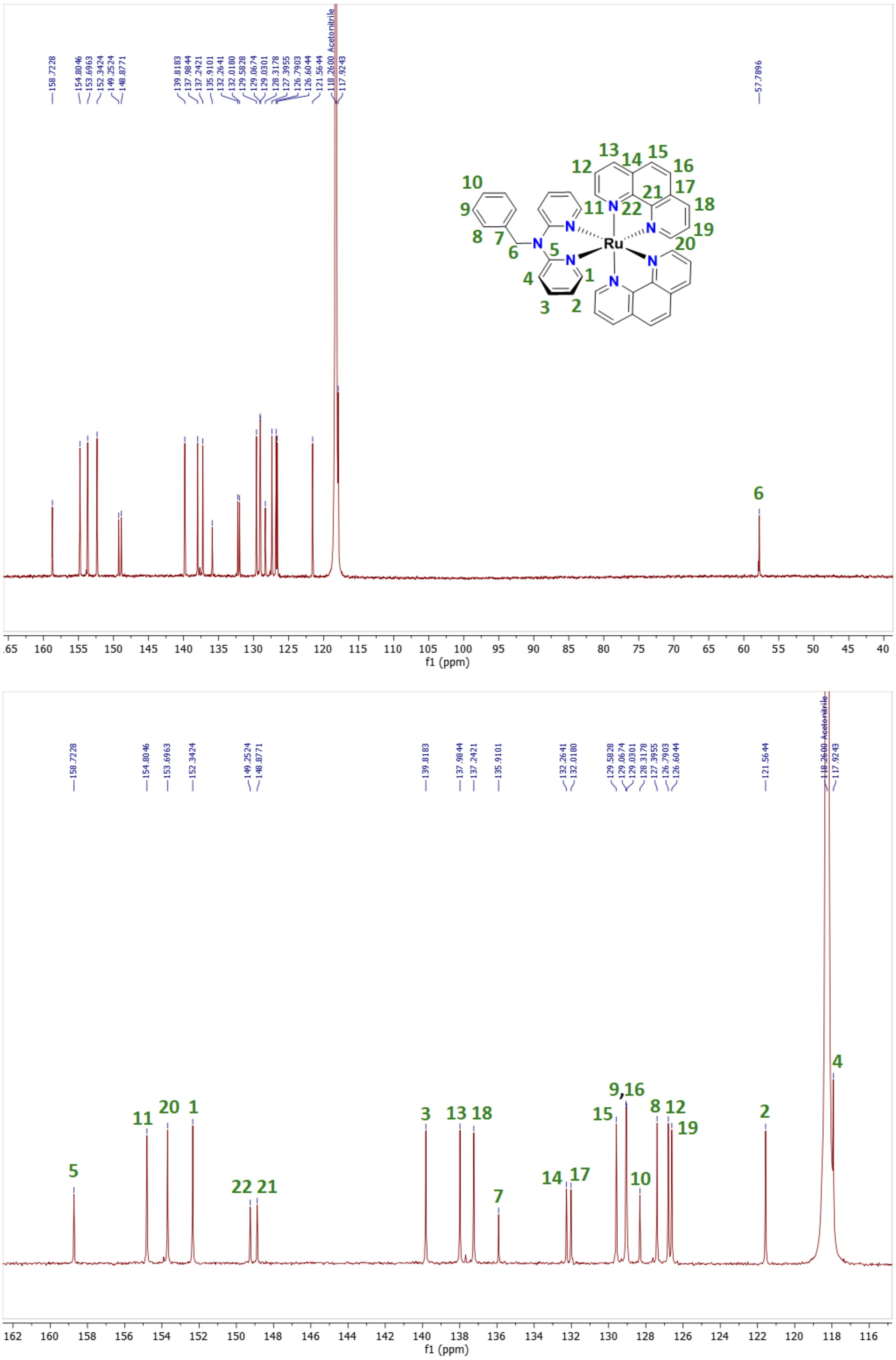


**Figure S12.** $^{13}$C NMR spectrum of [Ru(phen)$_2$**L2**](PF$_6$)$_2$ (**2**) in CD$_3$CN. Top: complete spectrum; bottom: zoom between 162 and 115 ppm).

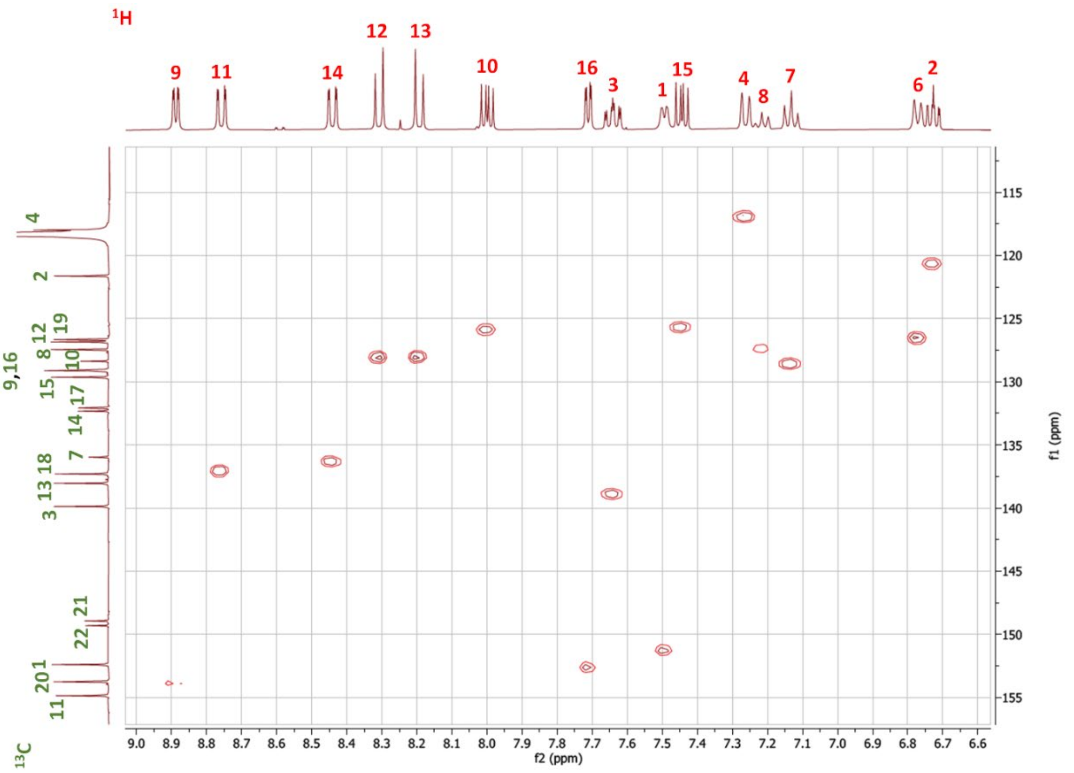


**Figure S13.** $^{1}$**H**-$^{13}$**C** HSQC NMR spectrum of [Ru(phen)$_2$**L2**](PF$_6$)$_2$ (**2**). The **H**- and **C**-atom numbering are shown in **Figures S10** and **S11**, respectively.

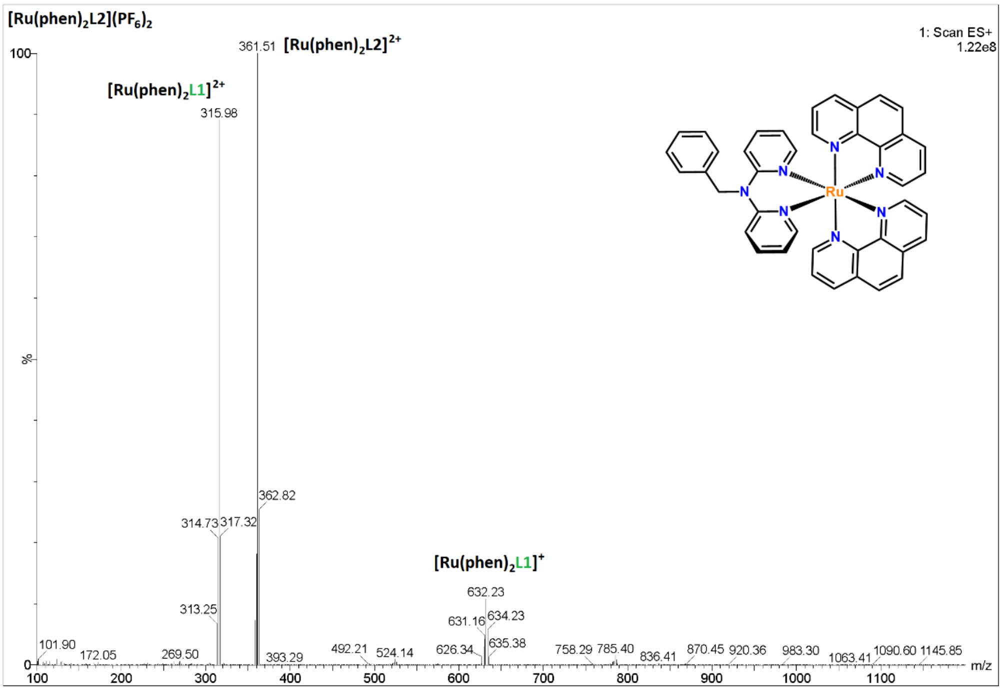


**Figure S14.** ESI-MS spectrum of complex [Ru(phen)$_2$**L2**](PF$_6$)$_2$ (**2**). LRMS (ES+) m/z [M]$^{2+}$ calcd. for $C_{41}H_{31}N_7Ru$, 361.41; m/z [M-Bn]$^{2+}$ calcd. for $C_{34}H_{24}N_7Ru$, 316.08; m/z [M-Bn]$^{+}$ calcd for $C_{34}H_{24}N_7Ru$, 631.69.

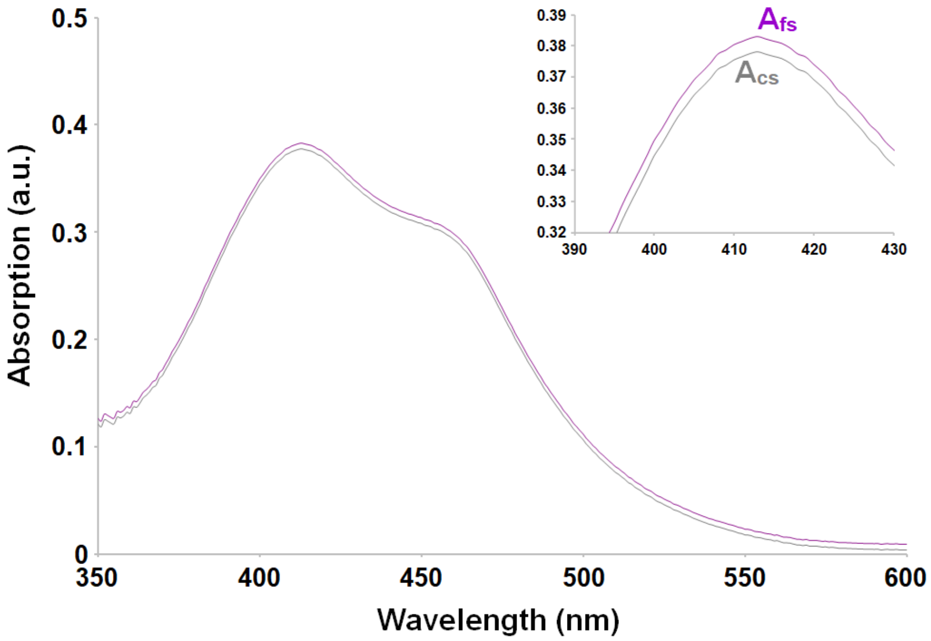


**Figure S15.** UV-Vis spectra used for the determination of log $P_{o/w}$ (lipophilic character) for complex **1**. The MLCT absorption band at λ = 413 nm was used for the calculations. Violet spectrum: absorption after partition in water saturated with octan-1-ol ($A_{fs}$); grey spectrum: absorption after subsequent partition in octan-1-ol saturated with water ($A_{cs}$) (see **Experimental Section** for details).

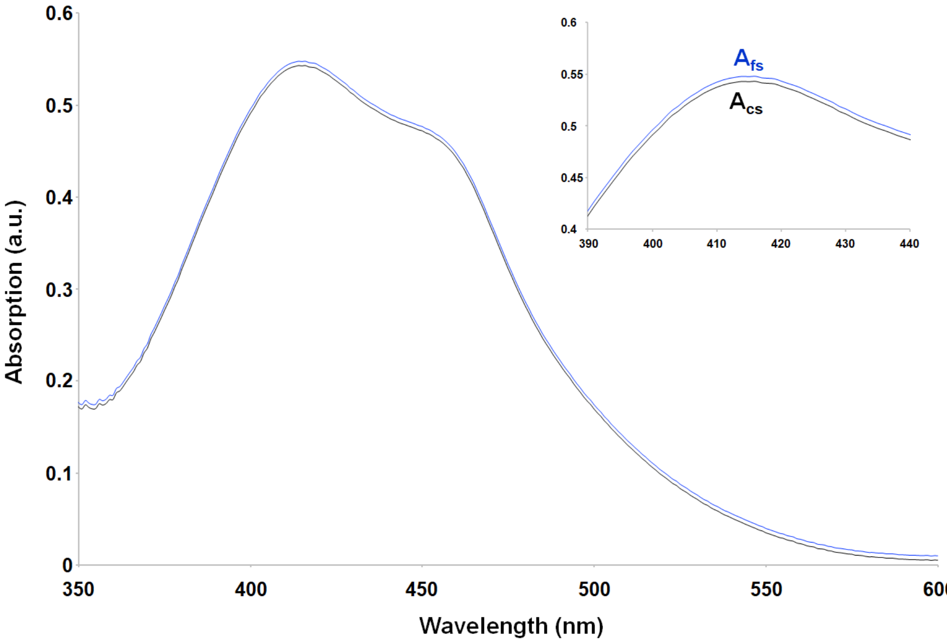


**Figure S16.** UV-Vis spectra used for the determination of log $P_{o/w}$ (lipophilic character) for complex **2**. The MLCT absorption band at λ = 414 nm was used for the calculations. Blue spectrum: absorption after partition in water saturated with octan-1-ol ($A_{fs}$); black spectrum: absorption after subsequent partition in octan-1-ol saturated with water ($A_{cs}$) (see **Experimental Section** for details).

**Table S1.** Evaluation of the lipophilic character of **1** and **2** using the shake-flask method. UV-Vis spectroscopic data ($A_{cs}$ and $A_{fs}$) obtained and corresponding calculated log $P_{o/w}$ values with their standard deviations, determined from three independent experiments.

| | 1 | | | 2 | | |
|---|---|---|---|---|---|---|
| | Exp 1 | Exp 2 | Exp 3 | Exp 1 | Exp 2 | Exp 3 |
| $A_{cs}$ | 0.376 | 0.366 | 0.386 | 0.542 | 0.551 | 0.542 |
| $A_{fs}$ | 0.380 | 0.369 | 0.390 | 0.547 | 0.556 | 0.546 |
| log $P_{o/w}$ | 1.93 | 1.94 | 1.91 | 2.01 | 2.04 | 2.07 |
| Average log $P_{o/w}$ | | 1.93 | | | 2.04 | |
| Std. deviation log $P_{o/w}$ | | 0.02 | | | 0.03 | |

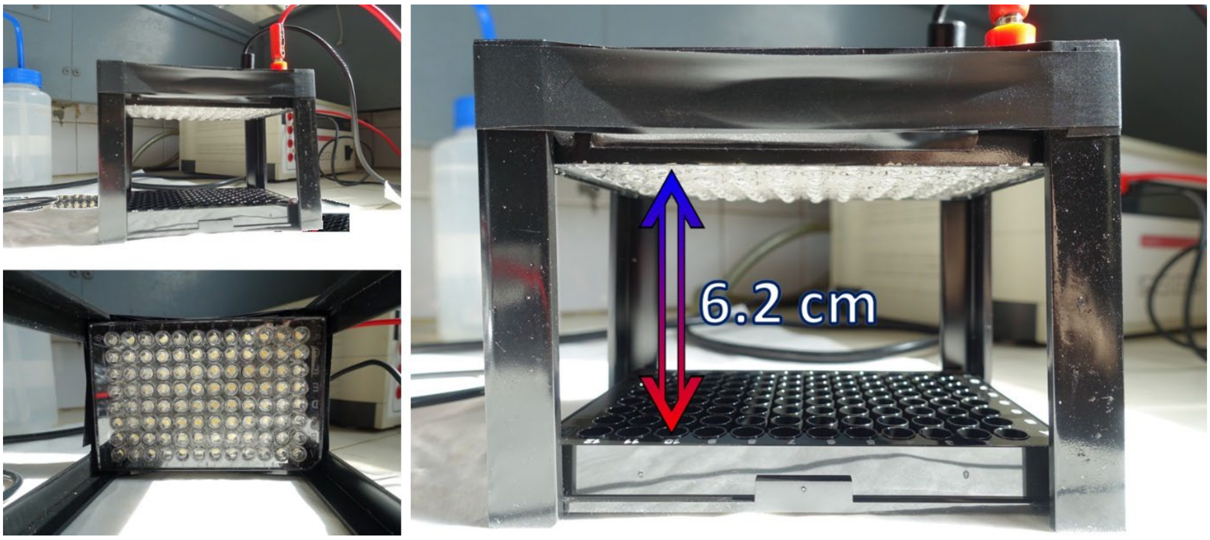


**Figure S17.** Various views of the homemade lamp including 96 LEDs (RND components, ref.: RND 135-00220 purchased from DISTRELEC) whose spectral distribution is shown in **Figure S18.** The distance between the LEDs and the wells (of the cell-culture plates) is of 6.2 cm.

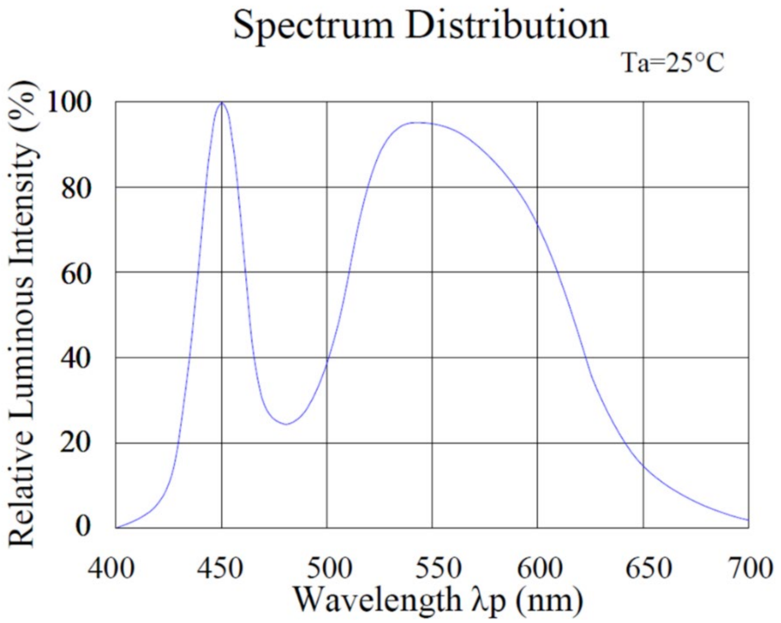


**Figure S18.** Spectral distribution of the white LEDs from RND components, (ref.: RND 135-00220 – DISTRELEC), which were used for the cytotoxicity studies.

**Table S2**. Crystal data and structure refinement for compound **2** (CCDC **2218777**).

| | |
|---|---|
| Empirical formula | $C_{43}H_{34}F_{12}N_8P_2Ru$ |
| Formula weight (g mol$^{-1}$) | 1053.79 |
| Temperature (K) | 296(2) |
| Crystal system | monoclinic |
| Space group | $P2_1/c$ |
| Crystal size (mm$^3$) | 0.20 × 0.25 × 0.40 |
| $a$ (Å) | 19.5843(8) |
| $b$ (Å) | 9.4572(4) |
| $c$ (Å) | 23.4404(10) |
| $\alpha$ (°) | 90 |
| $\beta$ (°) | 96.134(2) |
| $\gamma$ (°) | 90 |
| $V$ (Å$^3$) | 4316.6(3) |
| $Z$ | 4 |
| $\rho_{calcd}$ | 1.622 |
| $\mu$ (mm$^{-1}$) | 0.533 |
| $F$(000) | 2120 |
| $\theta$ for data collection (°) | 1.748–28.700 |
| Reflections collected / unique | 44716 / 11077 |
| Completeness to theta | 0.994 |
| Data / restraints / parameters | 11077 / 0 / 572 |
| Goodness-of-fit on $F^2$ | 1.050 |
| Final $R$ indices [$I$>2$\sigma$($I$)] | R1 = 0.0653, wR2 = 0.1884 |
| $R$ indices (all data) | R1 = 0.0858, wR2 = 0.2050 |
| largest diff. peak and hole ($e$ A$^3$) | 1.812 and –1.171 |

**Table S3.** Selected bond distances (Å) and angles (°) for compounds **1** and **2**. The atom labelling is shown in **Figure 1** (main text).

| | Compound **1**[1] | Compound **2** |
|---|---|---|
| Ru1–N1 | 2.056(3) | 2.074(3) |
| Ru1–N2 | 2.067(3) | 2.072(4) |
| Ru1–N3 | 2.056(3) | 2.059(4) |
| Ru1–N4 | 2.083(3) | 2.081(3) |
| Ru1–N5 | 2.088(3) | 2.088(3) |
| Ru1–N6 | 2.084(3) | 2.082(3) |
| | | |
| N1–Ru1–N2 | 80.11(11) | 79.59(13) |
| N1–Ru1–N3 | 94.47(11) | 94.28(13) |
| N1–Ru1–N4 | 171.28(11) | 171.26(13) |
| N1–Ru1–N5 | 96.51(10) | 93.80(12) |
| N1–Ru1–N6 | 88.74(11) | 90.42(13) |
| N2–Ru1–N3 | 84.88(10) | 85.17(14) |
| N2–Ru1–N4 | 92.67(10) | 93.42(14) |
| N2–Ru1–N5 | 174.99(10) | 172.56(14) |
| N2–Ru1–N6 | 96.63(11) | 97.93(13) |
| N3–Ru1–N4 | 79.92(10) | 79.81(14) |
| N3–Ru1–N5 | 91.74(10) | 91.96(14) |
| N3–Ru1–N6 | 176.65(11) | 174.79(13) |
| N4–Ru1–N5 | 90.36(10) | 92.83(14) |
| N4–Ru1–N6 | 97.00(10) | 95.78(13) |
| N5–Ru1–N6 | 86.94(11) | 85.45(12) |

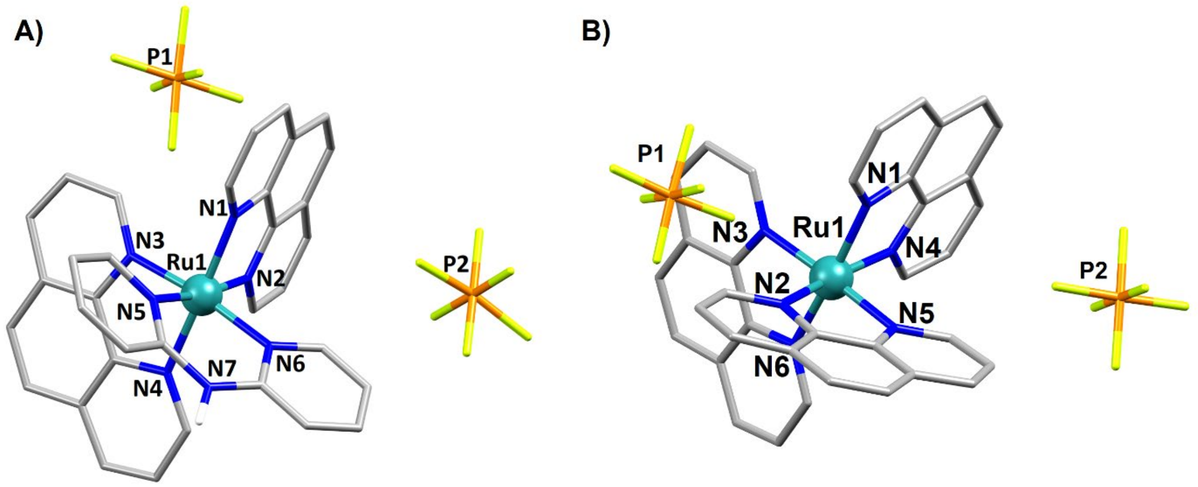


**Figure S19. A)** Representation of the crystal structure of [Ru(Phen)$_2$(dpa)](PF$_6$)$_2$.[1] **B)** Representation of the crystal structure of [Ru(Phen)$_3$](PF$_6$)$_2$.[2] The atoms coordinated to the metal centre and the metal ion are labelled. Hydrogen atoms are omitted for clarity.

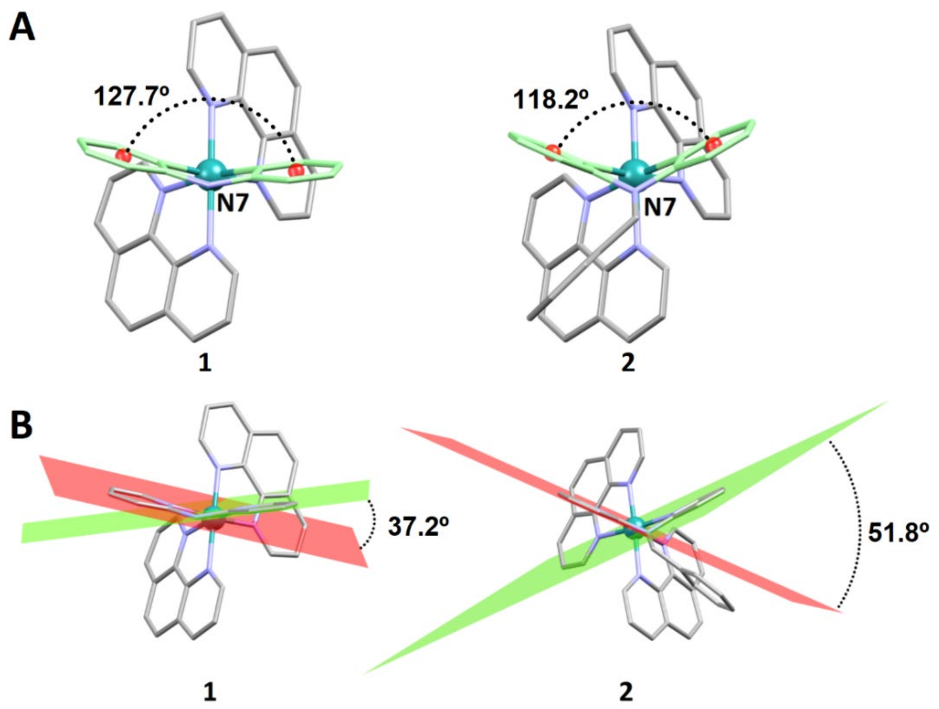


**Figure S20. A**) Angles between the two pyridine rings and the central nitrogen atom N7 of the coordinated dpa ligand in **1** (pyridine–N7–pyridine = 127.7°) and **2** (pyridine–N7–pyridine = 118.2°); **B**) angles between the planes containing the pyridine rings in **1** (angle = 37.2°) and **2** (angle = 51.8°).

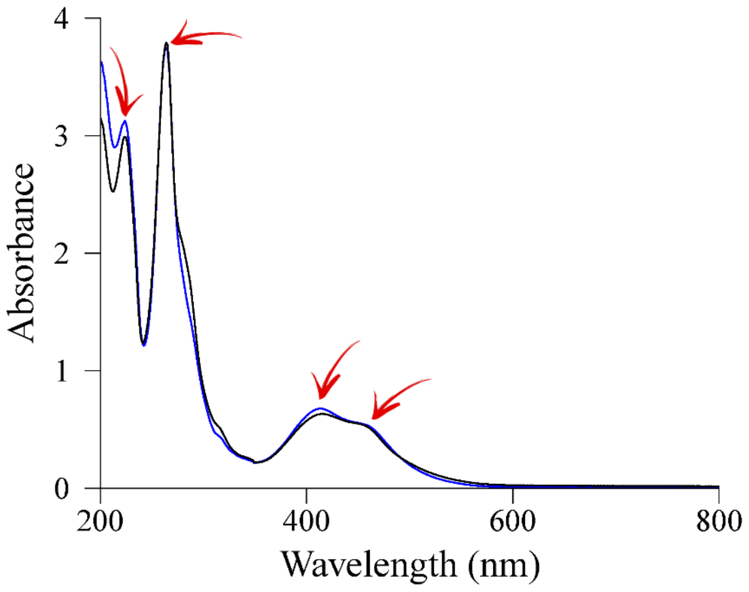


**Figure S21.** UV-Vis spectra of **1** (in black) and **2** (in blue) in water using a complex concentration of 50 μM.

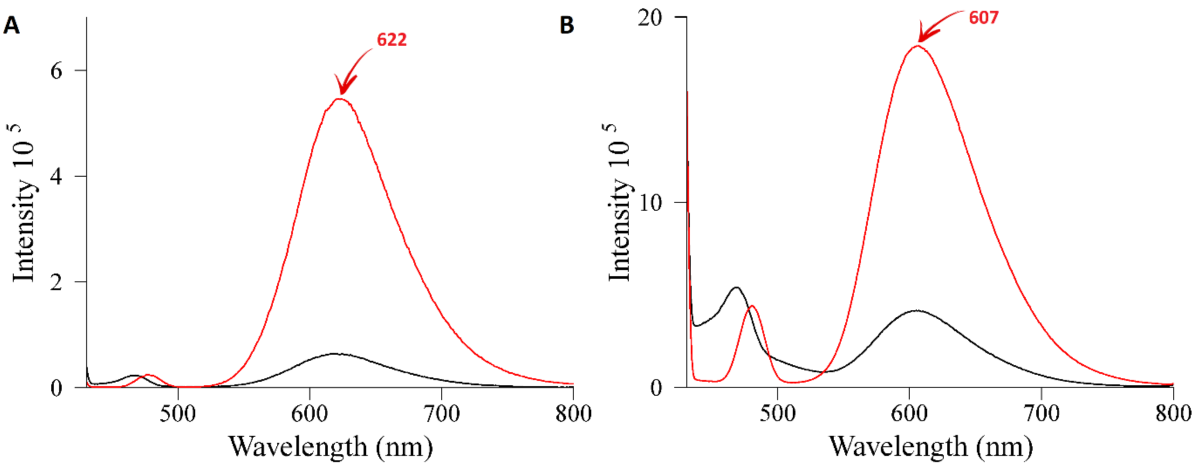


**Figure S22.** Fluorescence emission spectra of **A**) complex **1** in acetonitrile (black) and water (red) and **B**) complex **2** in acetonitrile (black) and water (red). [complex] = 5 µM; $\lambda_{exc}$ = 414 nm.

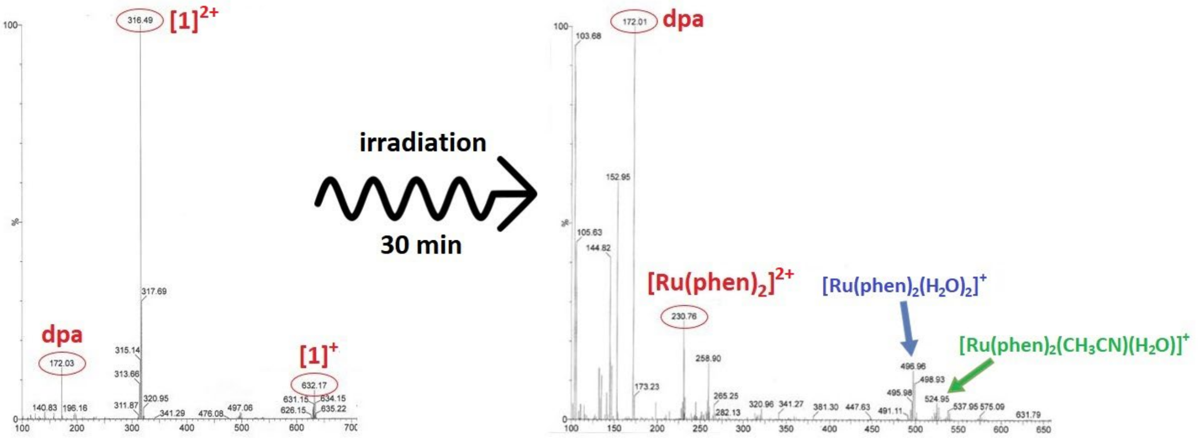


**Figure S23.** ESI-MS spectra of **1** before and after irradiation for 30 minutes with 1050-430 nm light. The irradiation was carried out with the complex dissolved in acetonitrile.

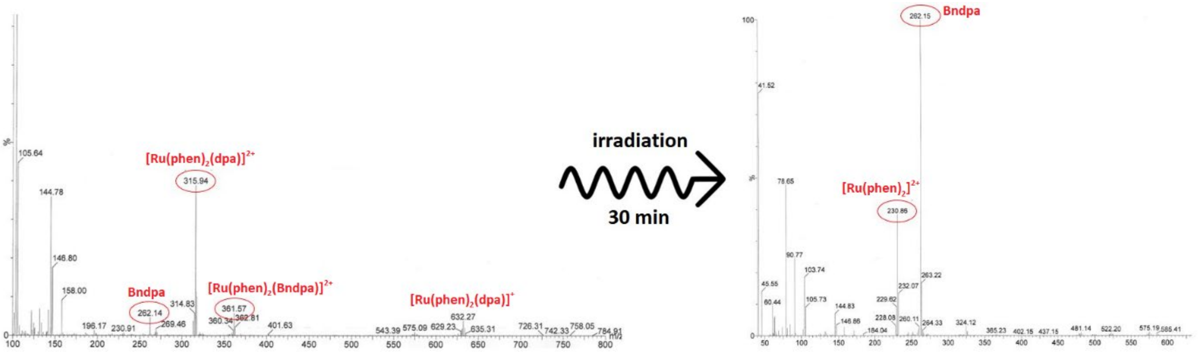


**Figure S24.** ESI-MS spectra of **2** before and after irradiation for 30 minutes with 1050-430 nm light. The irradiation was carried out with the complex dissolved in acetonitrile.

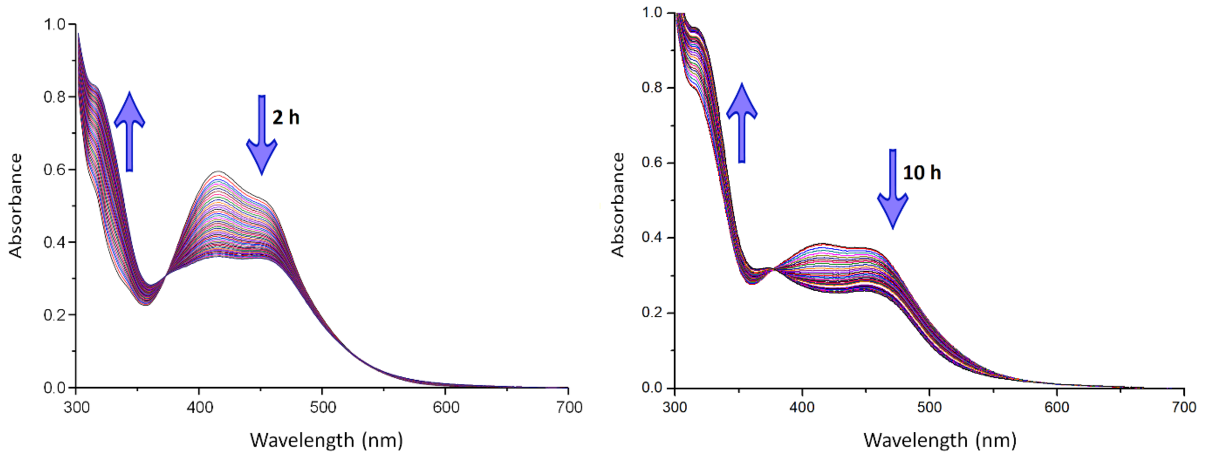


**Figure S25.** Time evolution of the UV/Vis data for complex **1** in *water*; [**1**] = 50 µM. Spectra were recorded at 25 °C every 100–120 seconds for up to 10 hours with continuous irradiation using 1050-430 nm light.

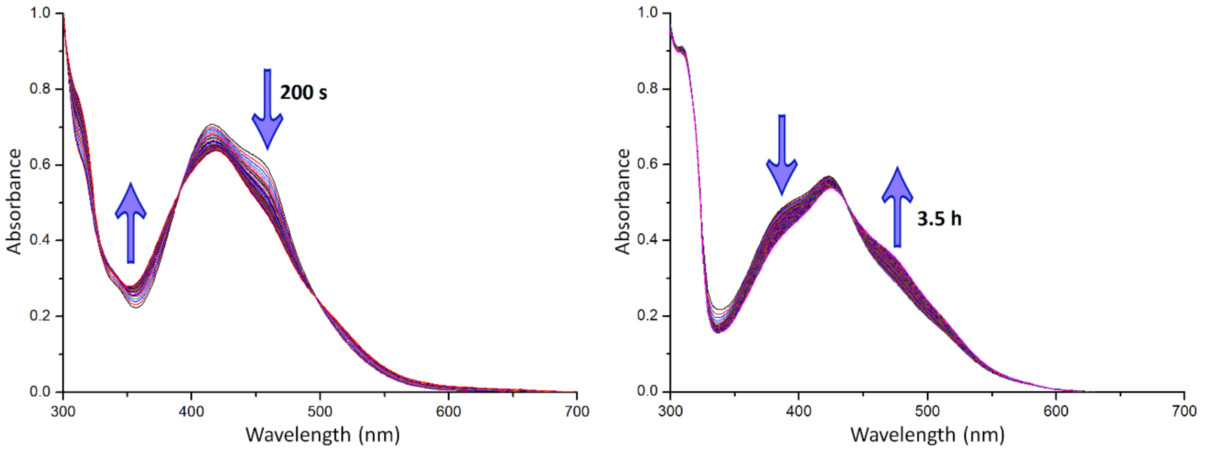


**Figure S26.** Time evolution of the UV/Vis data for complex **1** in *acetonitrile*; [**1**] = 50 µM. Spectra were recorded at 25 °C every 100–120 seconds for up to 10 hours with continuous irradiation using 1050-430 nm light.

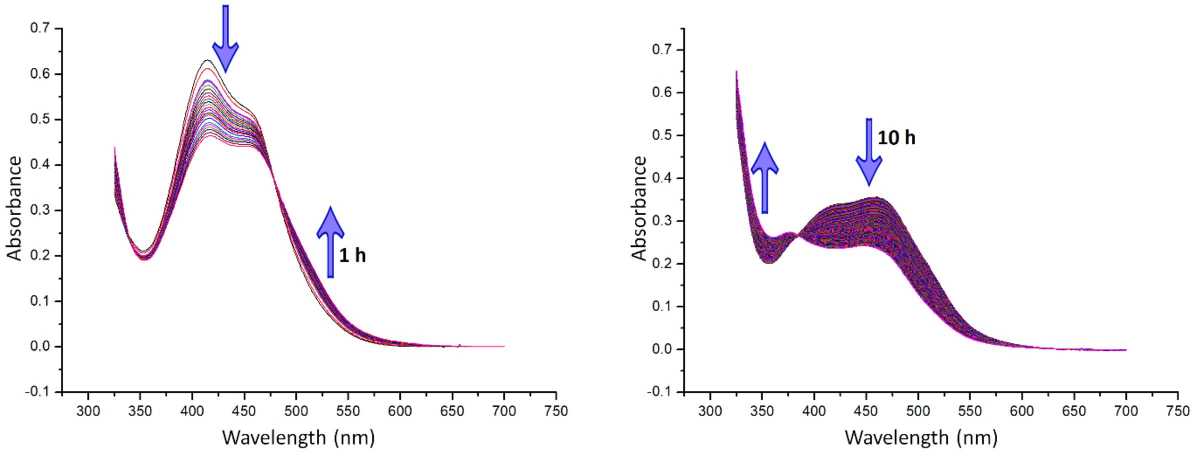


**Figure S27.** Time evolution of the UV/Vis data for complex **2** in *water*; [**2**] = 50 µM. Spectra were recorded at 25 °C every 100–120 seconds for up to 10 hours with continuous irradiation using 1050-430 nm light.

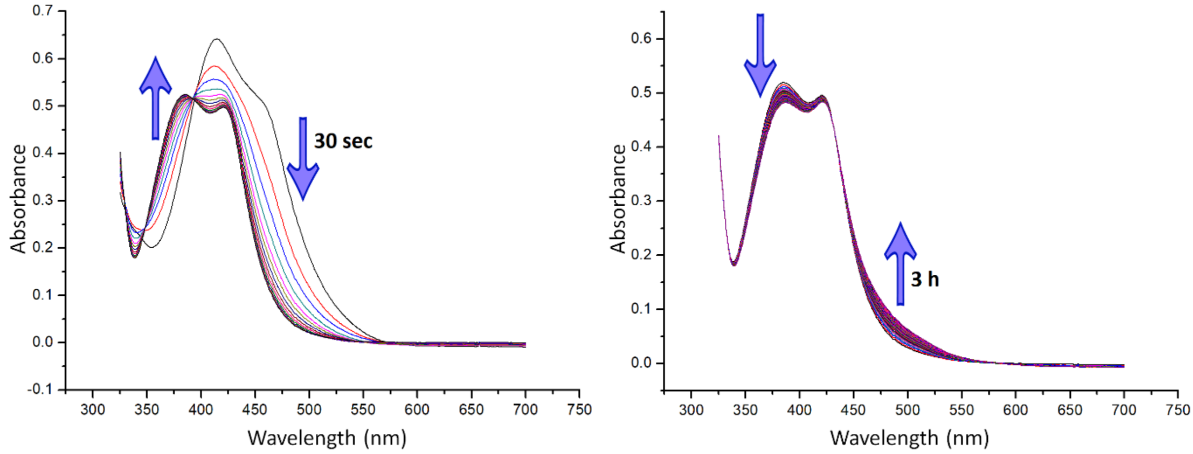


**Figure S28.** Time evolution of the UV/Vis data for complex **2** in *acetonitrile*; [**2**] = 50 µM. Spectra were recorded at 25 °C every 100–120 seconds for up to 10 hours with continuous irradiation using 1050-430 nm light.

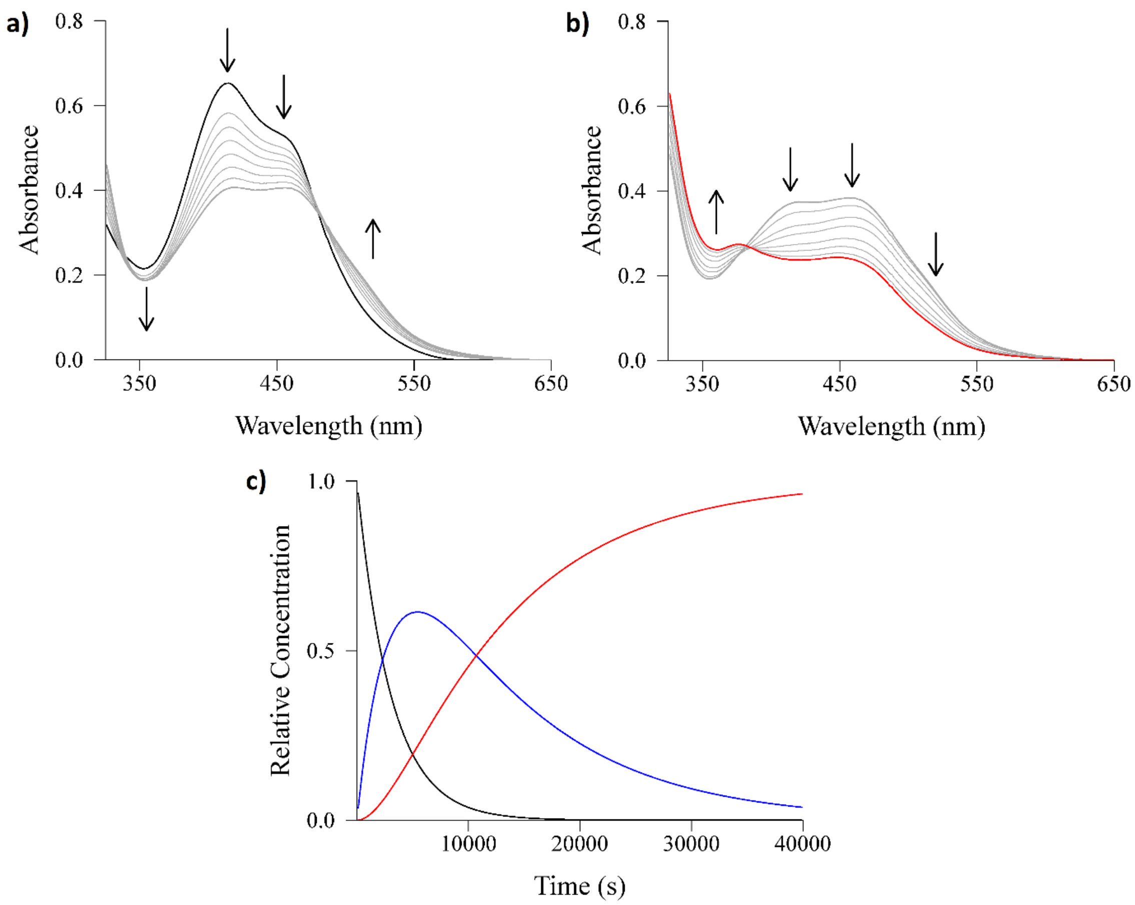


**Figure S29.** Time-resolved UV/Vis spectra of a 50 µM aqueous solution of **2** irradiated with 1050-430 nm light; a) step 1 with $t_{1/2}$ = 27 min, b) step 2 with $t_{1/2}$ = 123 min and c) relative concentrations of initial complex (black), intermediate species resulting from step 1 (blue) and the final complex (red) after step 2.

**Table S4.** $k_1$ and $k_2$ values (in $s^{-1}$) corresponding to respectively steps 1 and 2 in the photoinduced transformation process observed for **1** and **2** in water (various concentrations), water containing 10% DMSO and cacodylate buffer containing 10% DMSO.

| | Complex **1** | | Complex **2** | |
|---|---|---|---|---|
| Complex concentration and solvent | $k_1$ ($s^{-1}$) | $k_2$ ($s^{-1}$) | $k_1$ ($s^{-1}$) | $k_2$ ($s^{-1}$) |
| 5 µM water | - | - | $8.8\times10^{-4}$ | $1.2\times10^{-4}$ |
| 25 µM water | $3.2\times10^{-4}$ | $1.2\times10^{-4}$ | $7.0\times10^{-4}$ | $3.2\times10^{-4}$ |
| 10 µM water with 10% DMSO | - | - | $9.3\times10^{-4}$ | $1.3\times10^{-4}$ |
| 50 µM cacodylate with 10% DMSO | $3.8\times10^{-4}$ | $1.2\times10^{-4}$ | - | - |

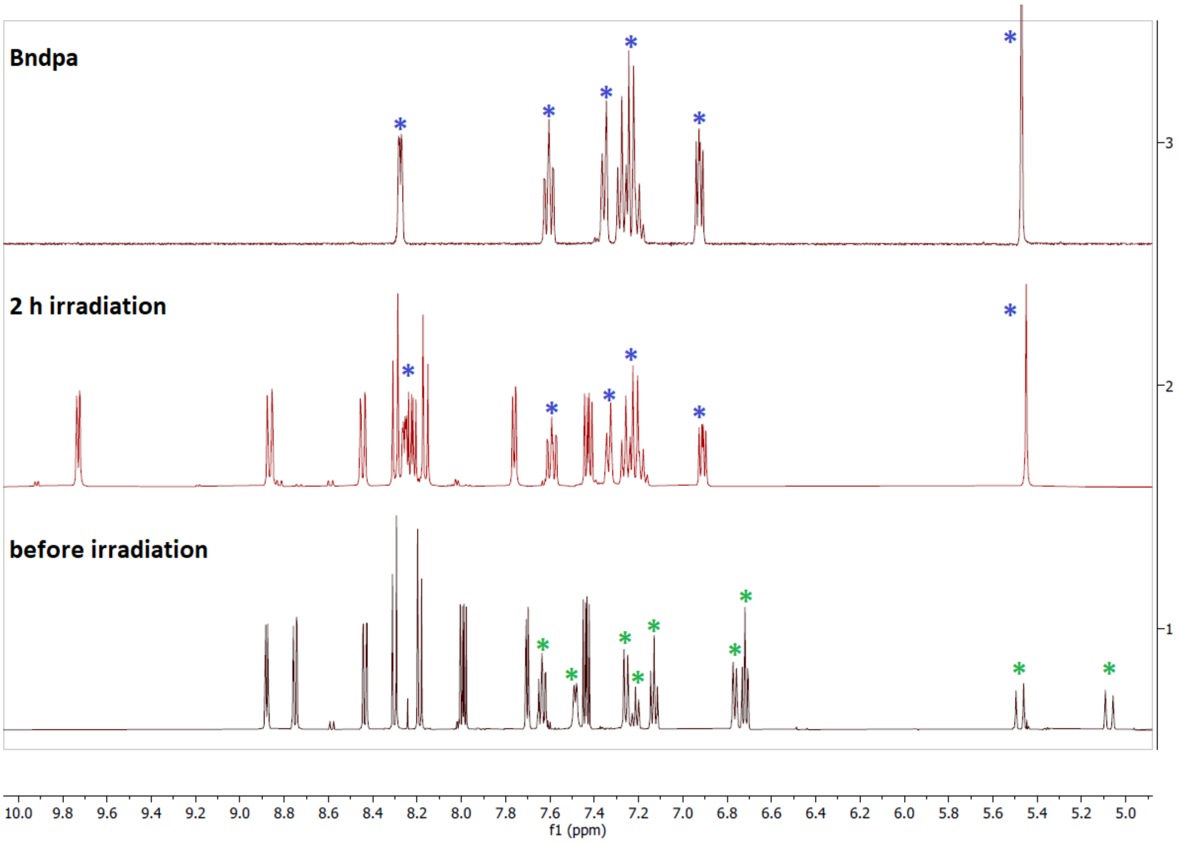


**Figure S30.** $^{1}H$ NMR spectra of free Bndpa in $CD_3CN$ (top), of complex **2** after 2 hours of irradiation with visible light (1050-430 nm) (middle) and of complex **2** before irradiation (bottom). The peaks marked with a green asterisk correspond to coordinated Bndpa and those with a blue asterisk to free Bndpa.

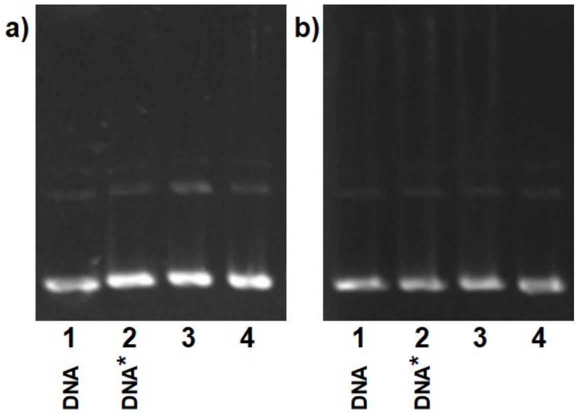


**Figure S31.** Agarose gel electrophoresis images of pBR322 plasmid DNA incubated with a) dpa and b) Bndpa. Lane 1: DNA, lane 2: DNA irradiated with 1050-430 nm light for 30 min (DNA*), lane 3: ligand, lane 4: ligand irradiated with 1050-430 nm light for 30 min.

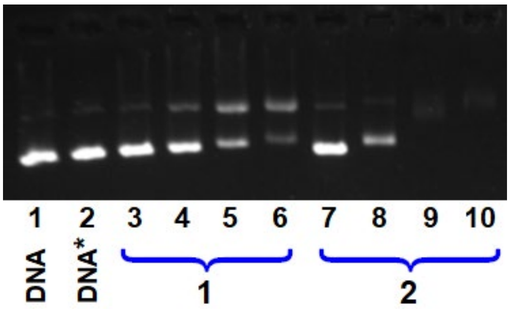


**Figure S32.** Agarose gel electrophoresis image of pBR322 plasmid DNA incubated with **1** and **2**, [complex] = 6.25 µM, applying increasing irradiation times (from 0 to 30 min) with 1050-430 nm light. Lane 1: DNA, lane 2: DNA irradiated for 30 min (DNA*), lanes 3–6: complex **1** irradiated for 0, 5, 15 and 30 min, lanes 7–10: complex **2** irradiated for 0, 5, 15 and 30 min. [DNA] = 15 µM (in base).

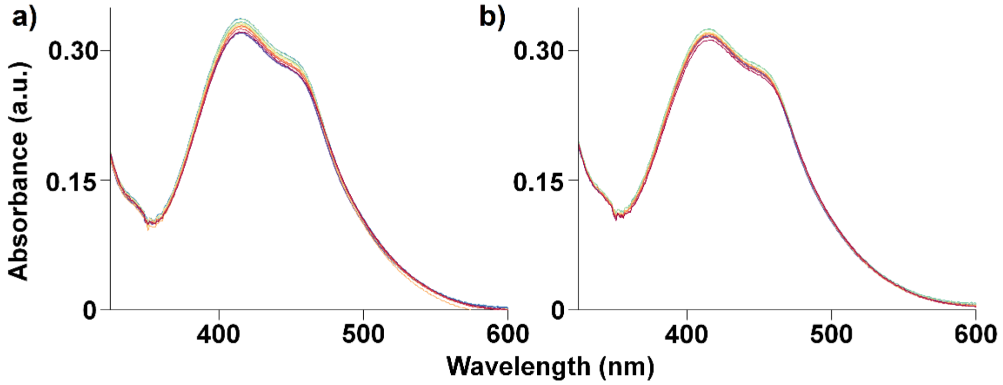


**Figure S33.** a) UV-Vis changes upon interaction of increasing amounts of DNA with **1**, namely using $[DNA]_{bp}$/[complex] of 0, 0.04, 0.11, 0.21, 0.32, 0.42, 0.53, 0.64, 0.85 and 1.06. [complex] = 25 µM; $[DNA]_{bp}$ = 0.2–12 µM. b) UV-Vis changes upon interaction of increasing amounts of DNA with **1** (same concentrations as before), which was pre-irradiated for 1 h.

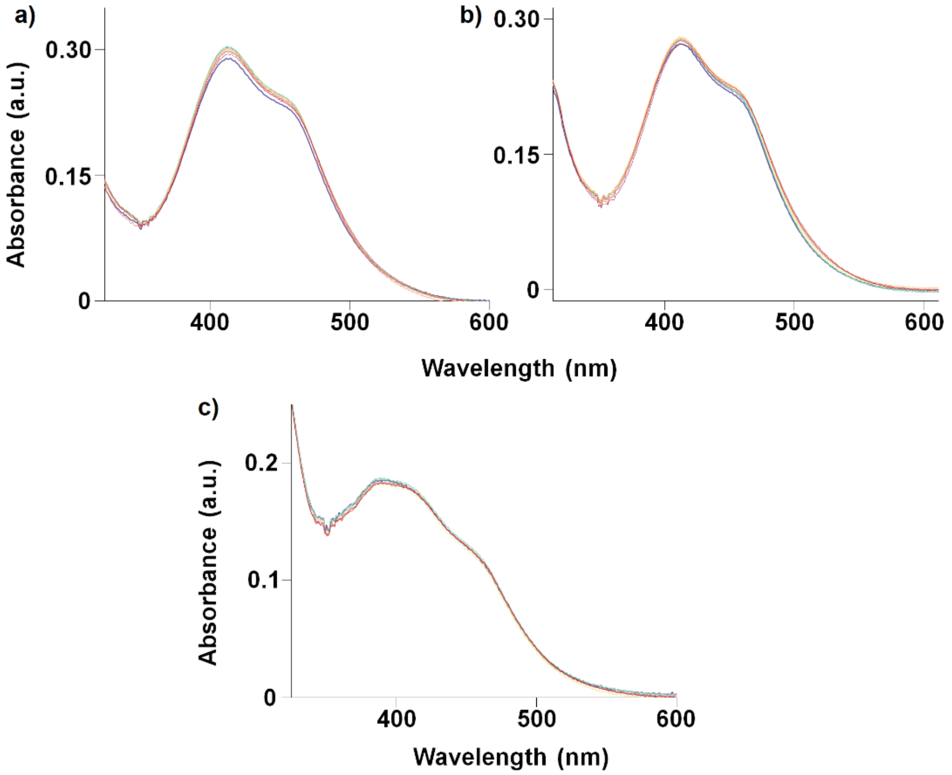


**Figure S34.** a) UV-Vis changes upon interaction of increasing amounts of DNA with **2**, namely using $[DNA]_{bp}$/[complex] ratios of 0, 0.04, 0.11, 0.21, 0.32, 0.42, 0.53, 0.64, 0.85 and 1.06. [complex] = 25 µM; [DNA] = 0.2–12 µM. b) UV-Vis changes upon interaction of increasing amounts of DNA with **2** (same concentrations as before), which was pre-irradiated for 1 h. c) UV-Vis spectra obtained with a solution of **2** that was pre-irradiated for 4 h (instead of 1 h).

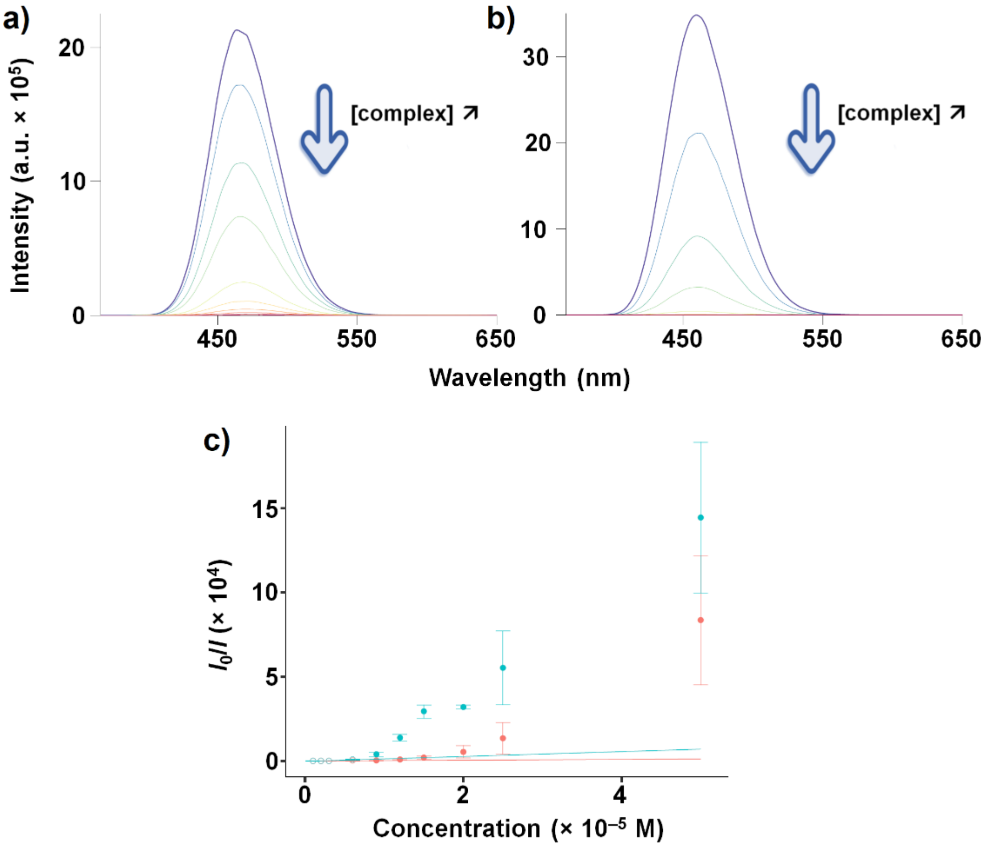


**Figure S35.** H3325 displacement assay with complex **1**, using [DNA] = 30 µM, [H3325] = 2 µM and increasing concentrations of complex, namely 0, 1 µM, 2 µM, 3 µM, 6 µM, 9 µM, 12 µM, 15 µM, 20 µM, 25 µM and 50 µM. a) Dark control; b) complex solution pre-irradiated for 4 h; c) Fitting of the fluorescence using the Stern-Volmer equation (dark control in red; irradiated complex in blue). Due to the non-linearity of the plots obtained, the $K_{SV}$ constants were determined at low [complex], *viz*. below 9 µM. All measurements were performed in duplicate.

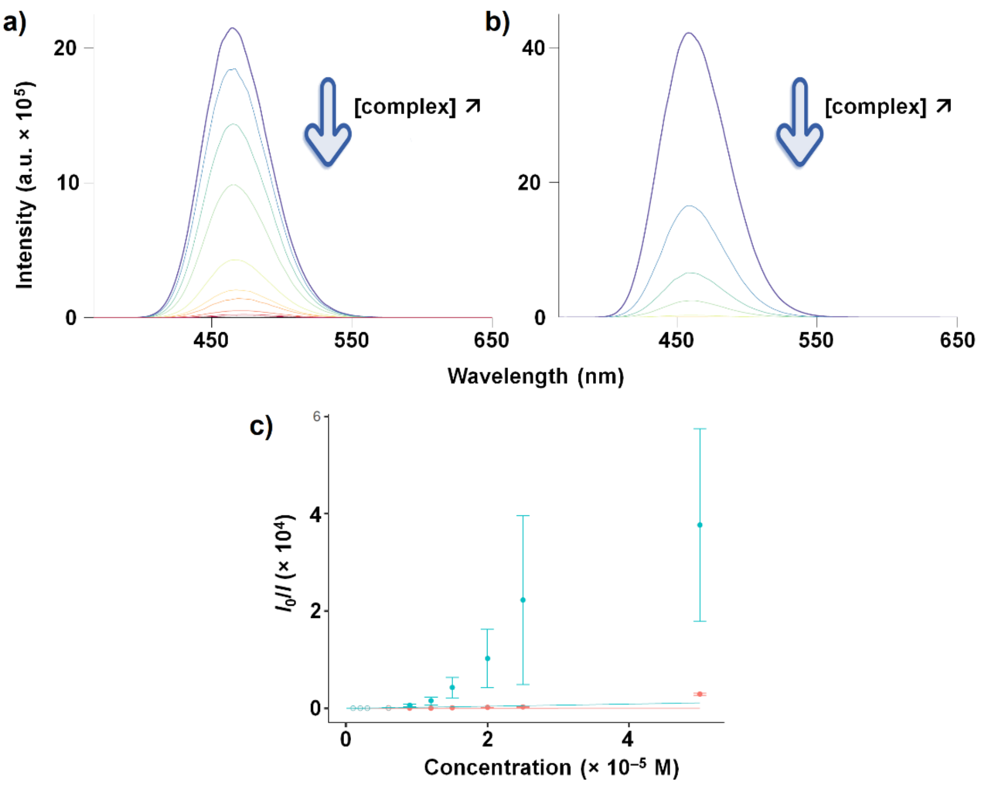


**Figure S36.** H3325 displacement assay with complex **2**, using $[DNA]_{bp}$ = 30 µM, [H3325] = 2 µM and increasing concentrations of complex, namely 0, 1 µM, 2 µM, 3 µM, 6 µM, 9 µM, 12 µM, 15 µM, 20 µM, 25 µM and 50 µM. a) Dark control; b) complex solution pre-irradiated for 4 h; c) Fitting of the fluorescence using the Stern-Volmer equation (dark control in red; irradiated complex in blue). Due to the non-linearity of the plots obtained, the $K_{SV}$ constants were determined at low [complex], *viz*. below 9 µM. All measurements were performed in duplicate.

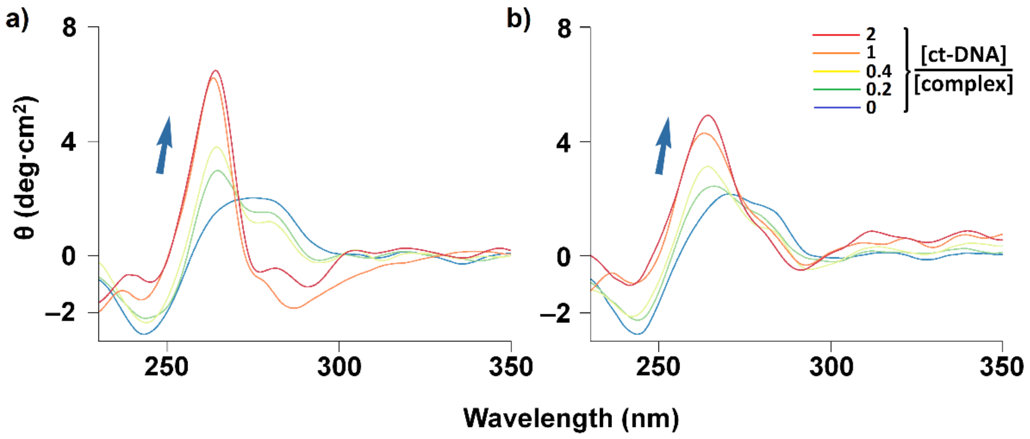


**Figure S37.** CD spectra of ct-DNA incubated with increasing amounts of complex **2**. [ct-DNA]/[complex] ratios = 0 (blue), 0.2 (green), 0.4 (yellow), 1 (orange) and 2 (red). [ct-DNA] = 50 µM. a) CD spectra recorded under dark conditions; b) CD spectra recorded using complex solutions that were pre-irradiated.

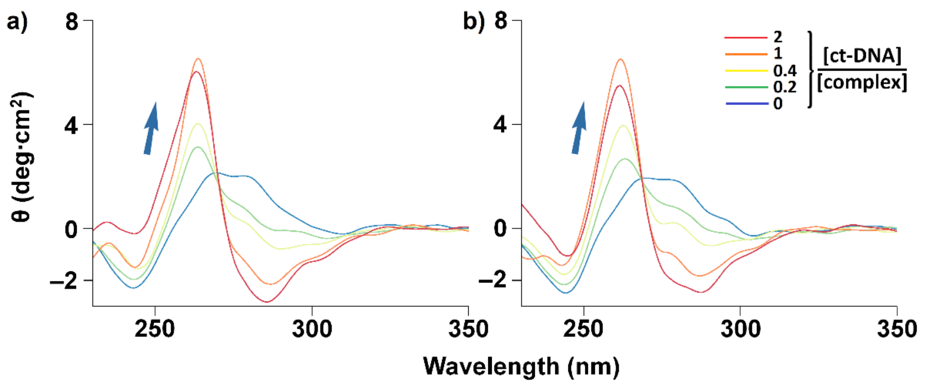


**Figure S38.** CD spectra of ct-DNA incubated for 24 h with increasing amounts of a) complex **1** and b) complex **2**. [ct-DNA]/[complex] ratios = 0 (blue), 0.2 (green), 0.4 (yellow), 1 (orange) and 2 (red). [ct-DNA] = 50 μM. a) CD spectra recorded under dark conditions; b) CD spectra recorded using complex solutions that were pre-irradiated.

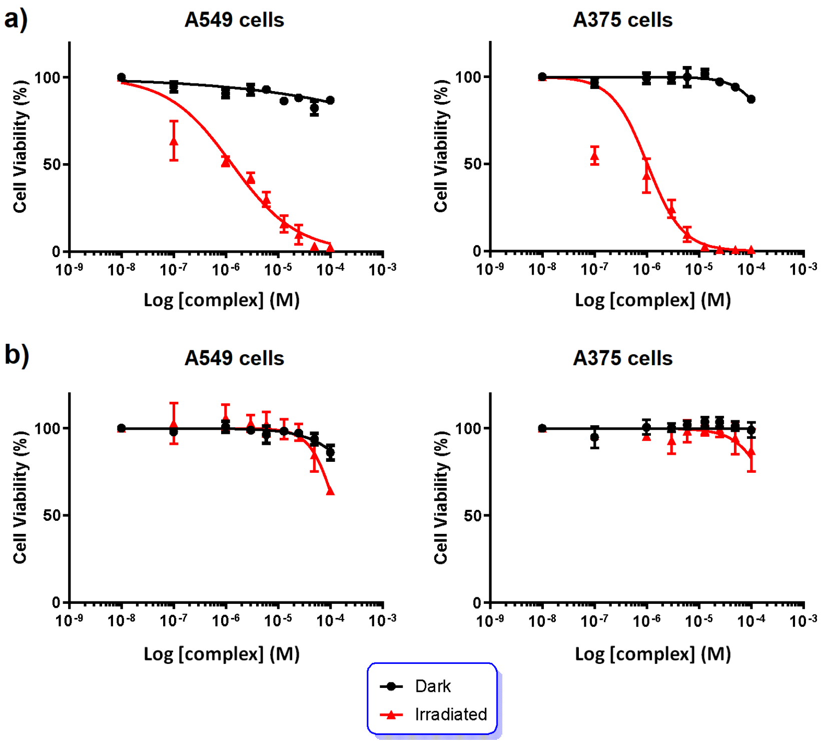


**Figure S39.** Dose-response curves (cell viability *versus* log [complex]) for a) complex **1** and b) complex **2** obtained with A549 cells (human lung carcinoma) and A375 cells (human melanoma). Drug exposure = 48 h; black dots = non-irradiated complex (dark conditions); red triangles = complex pre-irradiated for 1 h with 1050-430 nm light before incubation. All experiments (with double measurements for each sample) were carried out in triplicate.

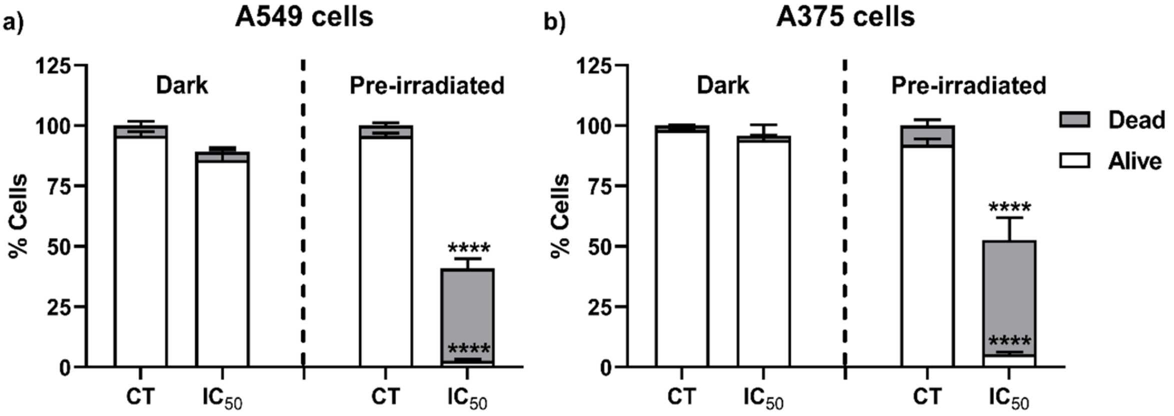


**Figure S40.** Analysis of cell death induced by compound **1** after Trypan blue staining. a) A549 and b) A375 cells were treated with the corresponding $IC_{50}$ values of compound **1** in both under dark and pre-irradiated conditions for 48 h. The mean ± standard errors are shown on the bars. The percentage of dying cells (blue stained cells) is illustrated in grey whereas the percentage of alive cells (cells not stained) is shown in white. The percentage of dying and alive cells of treated cells were normalized by their corresponding dark and pre-irradiated non-treated conditions (CT). Two-way ANOVA with Tukey *post*-hoc analysis was used to study the differences between treated and CT groups. Statistical differences are represented as **** $p < 0.0001$ treated *vs*. non-treated.

## References

1 M. G. B. Drew, S. Nag and D. Datta (2008) Inorganica Chimica Acta 361:417-421
2 J. Breu and A. J. Stoll (1996) Acta Crystallogr Sect C-Cryst Struct Commun 52:1174-1177
