# Supplementary material for: Steric hindrance, ligand ejection and associated photocytotoxic properties of ruthenium(II) polypyridyl complexes
Source: J Biol Inorg Chem. 2023 Apr 15;28(4):403–20. doi: 10.1007/s00775-023-01998-z (PMC10149480; doi:10.1007/s00775-023-01998-z)

checkCIF/PLATON report

Structure factors have been supplied for datablock(s) 2036sb15_0m

THIS REPORT IS FOR GUIDANCE ONLY. IF USED AS PART OF A REVIEW PROCEDURE FOR PUBLICATION, IT SHOULD NOT REPLACE THE EXPERTISE OF AN EXPERIENCED CRYSTALLOGRAPHIC REFEREE.

No syntax errors found. CIF dictionary Interpreting this report

Datablock: 2036sb15_0m

Bond precision:	C-C = 0.0075 A	Wavelength=0.71073	
Cell:	a=19.5843(8)	b=9.4572(4)	c=23.4404(10)
	alpha=90	beta=96.134(2)	gamma=90
Temperature:	296 K		

	Calculated	Reported
Volume	4316.6(3)	4316.6(3)
Space group	P 21/c	P 21/c
Hall group	-P 2ybc	-P 2ybc
Moiety formula	C41 H31 N7 Ru, 2(F6 P), C2 H3 N	?
Sum formula	C43 H34 F12 N8 P2 Ru	C43 H34 F12 N8 P2 Ru
Mr	1053.79	1053.79
Dx,g cm-3	1.622	1.622
Z	4	4
Mu (mm-1)	0.533	0.533
F000	2120.0	2120.0
F000'	2117.00	
h,k,lmax	26,12,31	26,12,31
Nref	11160	11077
Tmin,Tmax	0.852,0.899	0.658,0.746
Tmin'	0.808	

Correction method= # Reported T Limits: Tmin=0.658 Tmax=0.746
AbsCorr = MULTI-SCAN

Data completeness= 0.993 Theta(max)= 28.700

R(reflections)= 0.0653(8339) wR2(reflections)= 0.2050(11077)

S = 1.050 Npar= 572

The following ALERTS were generated. Each ALERT has the format
test-name_ALERT_alert-type_alert-level.
Click on the hyperlinks for more details of the test.

Alert level C

```
PLAT244_ALERT_4_C Low    'Solvent' Ueq as Compared to Neighbors of        C43 Check
PLAT250_ALERT_2_C Large U3/U1 Ratio for Average U(i,j) Tensor ....        2.3 Note
PLAT260_ALERT_2_C Large Average Ueq of Residue Including        P1      0.117 Check
PLAT260_ALERT_2_C Large Average Ueq of Residue Including        P2      0.155 Check
PLAT601_ALERT_2_C Unit Cell Contains Solvent Accessible VOIDS of .         32 Ang**3
PLAT906_ALERT_3_C Large K Value in the Analysis of Variance ......      2.895 Check
PLAT911_ALERT_3_C Missing FCF Refl Between Thmin & STh/L=    0.600         44 Report
PLAT918_ALERT_3_C Reflection(s) with I(obs) much Smaller I(calc) .          1 Check
PLAT971_ALERT_2_C Check Calcd Resid. Dens.  1.23Ang From F8              1.76 eA-3
PLAT971_ALERT_2_C Check Calcd Resid. Dens.  0.29Ang From F11             1.73 eA-3
PLAT971_ALERT_2_C Check Calcd Resid. Dens.  1.07Ang From F7              1.72 eA-3
```

Alert level G

```
PLAT002_ALERT_2_G Number of Distance or Angle Restraints on AtSite          4 Note
PLAT083_ALERT_2_G SHELXL Second Parameter in WGHT  Unusually Large       6.89 Why ?
PLAT171_ALERT_4_G The CIF-Embedded .res File Contains EADP Records          1 Report
PLAT173_ALERT_4_G The CIF-Embedded .res File Contains DANG Records          3 Report
PLAT231_ALERT_4_G Hirshfeld Test (Solvent)   P2       --F7        .      46.0 s.u.
PLAT231_ALERT_4_G Hirshfeld Test (Solvent)   P2       --F8        .      50.7 s.u.
PLAT231_ALERT_4_G Hirshfeld Test (Solvent)   P2       --F9        .      47.0 s.u.
PLAT231_ALERT_4_G Hirshfeld Test (Solvent)   P2       --F10       .      50.3 s.u.
PLAT231_ALERT_4_G Hirshfeld Test (Solvent)   P2       --F11       .      19.7 s.u.
PLAT244_ALERT_4_G Low    'Solvent' Ueq as Compared to Neighbors of         P1 Check
PLAT244_ALERT_4_G Low    'Solvent' Ueq as Compared to Neighbors of         P2 Check
PLAT790_ALERT_4_G Centre of Gravity not Within Unit Cell: Resd.  #          4 Note
              C2 H3 N
PLAT794_ALERT_5_G Tentative Bond Valency for Ru1       (III)      .      2.89 Info
PLAT860_ALERT_3_G Number of Least-Squares Restraints ............           3 Note
PLAT883_ALERT_1_G No Info/Value for _atom_sites_solution_primary .     Please Do !
PLAT910_ALERT_3_G Missing # of FCF Reflection(s) Below Theta(Min).          1 Note
PLAT912_ALERT_4_G Missing # of FCF Reflections Above STh/L=  0.600         38 Note
PLAT933_ALERT_2_G Number of HKL-OMIT Records in Embedded .res File          2 Note
PLAT941_ALERT_3_G Average HKL Measurement Multiplicity ..........         4.0 Low
PLAT978_ALERT_2_G Number C-C Bonds with Positive Residual Density.          2 Info
PLAT992_ALERT_5_G Repd & Actual _reflns_number_gt Values Differ by          6 Check
```

0 **ALERT level A** = Most likely a serious problem - resolve or explain
0 **ALERT level B** = A potentially serious problem, consider carefully
11 **ALERT level C** = Check. Ensure it is not caused by an omission or oversight
21 **ALERT level G** = General information/check it is not something unexpected

1 ALERT type 1 CIF construction/syntax error, inconsistent or missing data
11 ALERT type 2 Indicator that the structure model may be wrong or deficient
6 ALERT type 3 Indicator that the structure quality may be low
12 ALERT type 4 Improvement, methodology, query or suggestion
2 ALERT type 5 Informative message, check

It is advisable to attempt to resolve as many as possible of the alerts in all categories. Often the minor alerts point to easily fixed oversights, errors and omissions in your CIF or refinement strategy, so attention to these fine details can be worthwhile. In order to resolve some of the more serious problems it may be necessary to carry out additional measurements or structure refinements. However, the purpose of your study may justify the reported deviations and the more serious of these should normally be commented upon in the discussion or experimental section of a paper or in the "special_details" fields of the CIF. checkCIF was carefully designed to identify outliers and unusual parameters, but every test has its limitations and alerts that are not important in a particular case may appear. Conversely, the absence of alerts does not guarantee there are no aspects of the results needing attention. It is up to the individual to critically assess their own results and, if necessary, seek expert advice.

Publication of your CIF in IUCr journals

A basic structural check has been run on your CIF. These basic checks will be run on all CIFs submitted for publication in IUCr journals (*Acta Crystallographica*, *Journal of Applied Crystallography*, *Journal of Synchrotron Radiation*); however, if you intend to submit to *Acta Crystallographica Section C* or *E* or *IUCrData*, you should make sure that full publication checks are run on the final version of your CIF prior to submission.

Publication of your CIF in other journals

Please refer to the *Notes for Authors* of the relevant journal for any special instructions relating to CIF submission.

PLATON version of 12/09/2022; check.def file version of 09/08/2022

Datablock 2036sb15_0m - ellipsoid plot

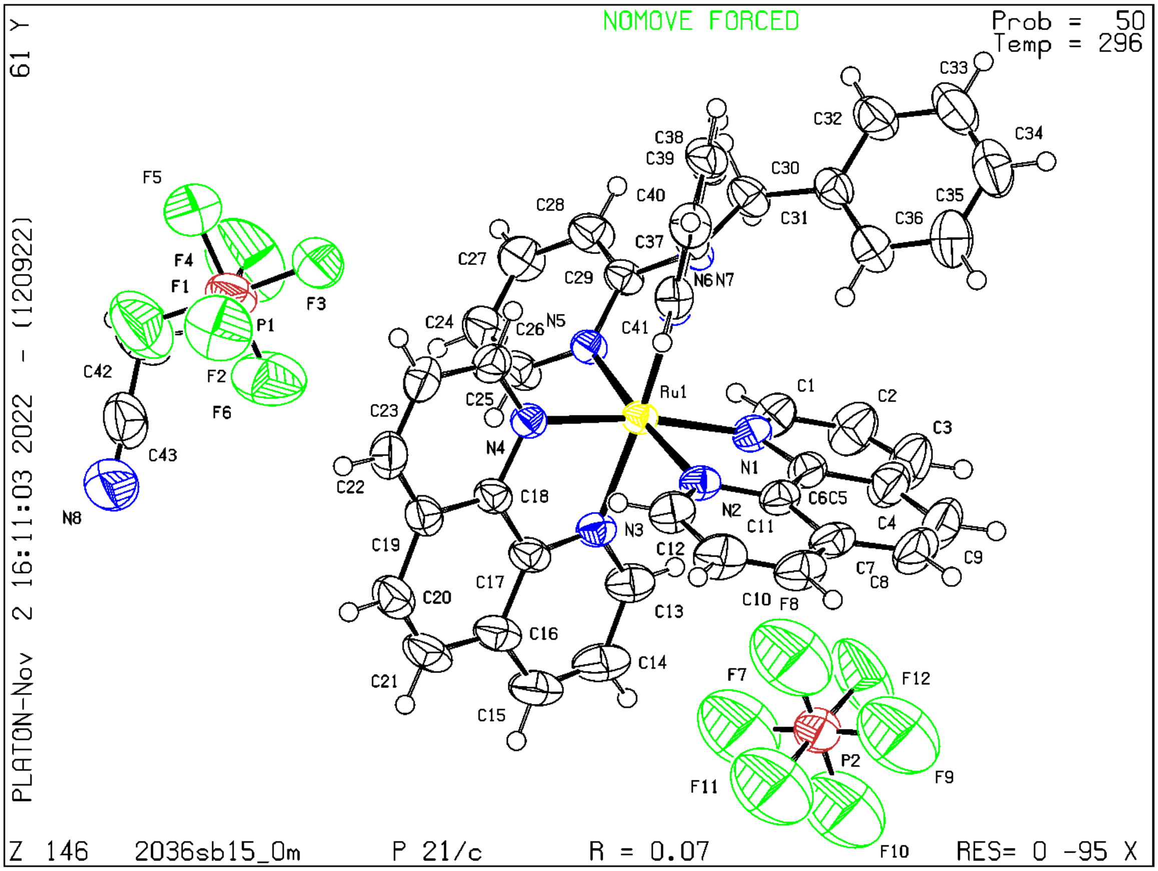

Supplement: Supplementary file 3 — (PDF 114 KB) [file 775_2023_1998_MOESM3_ESM.pdf]
